# Exploring Molecular Mechanisms of Paradoxical Activation in the BRAF Kinase Dimers: Atomistic Simulations of Conformational Dynamics and Modeling of Allosteric Communication Networks and Signaling Pathways

**DOI:** 10.1371/journal.pone.0166583

**Published:** 2016-11-18

**Authors:** Amanda Tse, Gennady M. Verkhivker

**Affiliations:** 1 Department of Computational and Data Sciences, Schmid College of Science and Technology, Chapman University, Orange, California, United States of America; 2 Chapman University School of Pharmacy, Irvine, California, United States of America; Wake Forest University, UNITED STATES

## Abstract

The recent studies have revealed that most BRAF inhibitors can paradoxically induce kinase activation by promoting dimerization and enzyme transactivation. Despite rapidly growing number of structural and functional studies about the BRAF dimer complexes, the molecular basis of paradoxical activation phenomenon is poorly understood and remains largely hypothetical. In this work, we have explored the relationships between inhibitor binding, protein dynamics and allosteric signaling in the BRAF dimers using a network-centric approach. Using this theoretical framework, we have combined molecular dynamics simulations with coevolutionary analysis and modeling of the residue interaction networks to determine molecular determinants of paradoxical activation. We have investigated functional effects produced by paradox inducer inhibitors PLX4720, Dabrafenib, Vemurafenib and a paradox breaker inhibitor PLX7904. Functional dynamics and binding free energy analyses of the BRAF dimer complexes have suggested that negative cooperativity effect and dimer-promoting potential of the inhibitors could be important drivers of paradoxical activation. We have introduced a protein structure network model in which coevolutionary residue dependencies and dynamic maps of residue correlations are integrated in the construction and analysis of the residue interaction networks. The results have shown that coevolutionary residues in the BRAF structures could assemble into independent structural modules and form a global interaction network that may promote dimerization. We have also found that BRAF inhibitors could modulate centrality and communication propensities of global mediating centers in the residue interaction networks. By simulating allosteric communication pathways in the BRAF structures, we have determined that paradox inducer and breaker inhibitors may activate specific signaling routes that correlate with the extent of paradoxical activation. While paradox inducer inhibitors may facilitate a rapid and efficient communication via an optimal single pathway, the paradox breaker may induce a broader ensemble of suboptimal and less efficient communication routes. The central finding of our study is that paradox breaker PLX7904 could mimic structural, dynamic and network features of the inactive BRAF-WT monomer that may be required for evading paradoxical activation. The results of this study rationalize the existing structure-functional experiments by offering a network-centric rationale of the paradoxical activation phenomenon. We argue that BRAF inhibitors that amplify dynamic features of the inactive BRAF-WT monomer and intervene with the allosteric interaction networks may serve as effective paradox breakers in cellular environment.

## Introduction

The human protein kinases are involved in regulation of many functional processes in signal transduction networks and represent one of the largest classes of clinically important therapeutic targets [**1–10**]. Protein kinases act as versatile activators and dynamic regulatory switches that are essential for regulation of cell cycle and organism development. A staggering amount of structural, genetic, and biochemical data on protein kinase genes has been accumulated in recent years, revealing a large variety of regulatory mechanisms, ranging from phosphorylation of kinase activation loops and autoinhibition to allosteric activation induced by dimerization or protein binding [**11–17]**. The steadily growing structural knowledge about conformational states of the kinase catalytic domain, regulatory assemblies, and kinase complexes with small molecule inhibitors has provided compelling evidence that conformational transformations between the inactive and active kinase states are central to the enzyme regulation and function [**18, 19**]. Functional conformational changes in protein kinases are operated by several regulatory regions of the catalytic domain: the conserved catalytic triad His-Arg-Asp (HRD), the DFG-Asp motif, the regulatory αC-helix, and the activation loop (A-loop). The inactive kinase states are often characterized by the DFG-out and closed A-loop conformations, while the active kinase forms feature the DFG-in and open A-loop conformations [**20–24**]. These regions are also involved in the formation of the regulatory spine (R-spine) and catalytic spine (C-spine) networks that are assembled and stabilized during conformational transformations to the active kinase states [**23,24**]. Despite diversity of regulatory mechanisms, modulation of kinase activity through dimerization and conformational repositioning of the αC-helix emerged as a common mechanism shared by several important protein kinase families, including ErbB kinases [**25–30**] and BRAF kinases [**31–37**]. Structural determinants of dimerization-induced regulation in the ErbB and BRAF kinases are rather similar, as the “off-state” of both enzymes is defined by a nonproductive αC-helix-out conformation supported by the presence of a short helical element in their A-loops that locks the enzyme in the inactive dormant form. Dimerization-induced allosteric regulation involves coordinated transitions of the kinase domain from the inactive monomer structure to a dimer configuration in which the αC-helix moves to an active ‘in’ conformation that ensures a productive alignment of the hydrophobic spines and catalytic residues required for activation. While a head-to-tail dimer assembly of the catalytic domains is characteristic of the ErbB kinases [**25–30**], a symmetric side-to-side dimer arrangement represents structural ‘modus operandi’ of the BRAF kinase activation [**31–37**]. The crystal structure of the inactive BRAF kinase has revealed a non-productive monomeric state of the enzyme, in which the αC-helix-out conformation can disrupt structural environment of the catalytic and regulatory residues near the ATP-binding site that is required for activation [**38]**. Dimer-inducing BRAF inhibitors irrespective of their binding modes may restrict the inter-lobe dynamics of the catalytic domains and promote stabilization of the active kinase conformations that facilitate the effective side-to-side dimerization [**39**]. Curbing the initial enthusiasm of the BRAF drug discovery efforts, the recent breakthrough studies have revealed that most of the existing BRAF inhibitors can paradoxically activate the wild-type (WT) BRAF kinase by inducing conformational changes and promoting dimerization that triggers RAS-independent transactivation and leads to MEK/ERK phosphorylation **[40–43].** The ability of dimer-inducing BRAF modulators to effectively inhibit the first monomer while conferring an active form of the second monomer may inadvertently stimulate kinase activation and promote ERK signaling [**44**]. As a result, therapeutic and clinical utility of BRAF inhibitors that exert paradoxical activation can be diminished.

**Fig 1:**
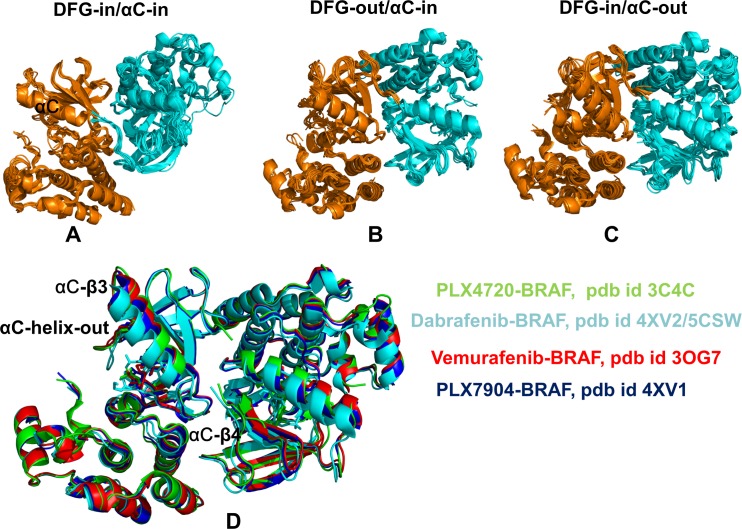
Structural Landscape of the BRAF Kinase Dimer Complexes with Small Molecule Inhibitors. (A) Structural alignment of the crystal structures of the BRAF kinase dimers with type I inhibitors (DFG-in/αC-in kinase conformation) are shown in green (monomer A) and cyan (monomer B) and included pdb entries 2FB8, 3D4Q, 3PSB, 3PRF, 3PRI, 3PPK, 3PPJ, 3Q4C, 3PSD, 4E26, 4H58, 4MNF, and 3OMV. (B) The structures of BRAF complexes with type II inhibitors (DFG-out/αC-in kinase conformation) included pdb entries 1UWH, 1UWJ, 5V9C, 5CT7, 4KSP, 4KSQ, 4FC0, 4G9R, 4G9C, 4DBN, 3Q96, 3II5, 3IDP, and 4JVG. (C) The crystal structures of BRAF complexes with type I½ inhibitors (DFG-in/αC-out kinase conformation) included pdb entries 3C4C, 3OG7, 4FK3, 4EHG, 3SKC, 3TV4, 3TV6, 4E4X, 4EHE, 4MBJ, 4PP7, 4CQE, 4XV1, 4XV2, 4XV3, 4XV9. In this class of BRAF complexes, some crystal structures (pdb entries 3SKC, 3TV4, 3TV6, 4E4X, 4EHE, 4MBJ, 4PP7) have a small helical motif in the activation segment. (D) The superposition of the crystal structures of BRAF dime complexes with PLX4720 (pdb id 3C4C) (in green), Vemurafenib (pdb id 3OG7) (in red), PLX7904 (pdb id 4XV1) (in blue), and Dabrafenib (pdb id 4XV2, 5CSW) (in cyan). The regulatory regions are annotated and structural arrangements of the αC-helix and the DFG motif in BRAF complexes are highlighted.

Based on the crystallographic binding mode, kinase inhibitors have been classified in several distinct classes [**45–50**]. Type I kinase inhibitors target the catalytically competent, active (DFG-in/ αC-helix-in) conformation of the kinase domain. Type II kinase inhibitors recognize the inactive DFG-out/αC-helix-in kinase state, while type I½ inhibitors often bind to another inactive DFG-in/αC-helix-out conformation that precludes the formation of the active enzyme form. Type II and type I½ kinase inhibitors often exhibit a higher degree of selectivity as these molecules bind to specific inactive conformations that may be structurally unique for a given kinase target. Many type I BRAF inhibitors have been developed and the crystal structures of their complexes with the BRAF dimers have been solved (**Fig 1**), including SB-590885 (pdb id 2FB8) [**51**] and GDC-0879 (pdb id 4MNF) [**52**]. Although type I BRAF inhibitors are effective against the oncogenic BRAF-V600E mutant, these molecules are usually strong dimer-promoting agents causing paradoxical activation in the BRAF-WT cells. Type II BRAF inhibitors [**53–58**] that stabilize the inactive DFG-out/αC-helix-in conformation can also stimulate enzyme transactivation by favoring an αC-helix-in position that promotes side-to-side dimerization (**Fig 1**). The crystal structures of the BRAF-WT and BRAF-V600E complexes with the FDA-approved Sorafenib (pdb id 1UWH, 1UWJ) have demonstrated that type II inhibitors that strongly bind to both monomers and exhibit long residence time may be relatively weak dimer inducers [**39**]. Another dimer-inducing type II inhibitor LY3009120 (pdb id 5C9C) is a pan-RAF inhibitor of all three RAF isoforms that causes only minimal paradoxical activation due to structural occupation and effective inhibition of both monomers [**54**]. Other notable type II BRAF inhibitors include TAK-632 [**55, 56**], RAF-625 which is now advanced into clinical trials [**57**], and AZ-628 [**58**]. Structural studies of the type II BRAF agents have demonstrated that their inhibitory effects may not directly correlate with a dimer-promoting potential and induction of paradoxical activation.

**Fig 2:**
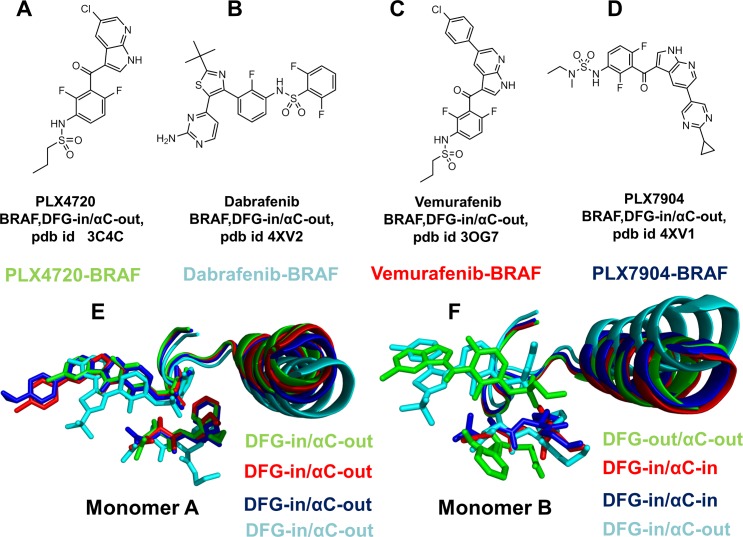
Chemical Structures and Structural Binding Modes of the BRAF Inhibitors. Chemical structures of studied BRAF inhibitors: (A) PLX4720, (B) Vemurafenib (B), PLX7904 (C) and Dabrafenib (D). (E) The binding modes of the BRAF inhibitors in the first binding site (monomer A). PLX4720 (in green), Vemurafenib (in red), PLX7904 (in blue), and Dabrafenib (in cyan). (F) The binding modes of the BRAF inhibitors in the second binding sites (monomer B). Among studied inhibitors, only PLX4720 (in green) and Dabrafenib (in cyan) bind to the second monomer. Note structural similarity of the inhibitor binding mode and DFG-in/αC-out kinase conformation in the first monomer, while alternative inhibitor binding modes and conformational variability in the second monomer.

Type I½ BRAF inhibitors target specific DFG-in/αC-helix-out kinase conformations and have exploited structural features of the inactive BRAF-WT monomer with a nonproductive αC-helix-out position [**59–64**]. Several BRAF structures complexed with type I½ inhibitors feature a small helical segment in the A-loop, which is present in the crystal structure of the inactive BRAF-WT monomer [**38**]. This class of BRAF inhibitors has offered unique opportunities for targeted modulation of the dimerization mechanism by sequestering specific kinase conformations with αC-helix-out arrangements that compromise the fidelity of the dimerization interface and may therefore preclude effective signaling. The crystal structure of the BRAF complex with type I½ inhibitor PLX4720 (pdb id 3C4C) has revealed an asymmetric dimer, in which one of the two binding sites was preferentially occupied by the inhibitor targeting the DFG-in/αC-helix-out kinase conformation, while the inhibitor adopts a different binding mode and binds to a DFG-out/αC-helix-out conformation in the second binding site [**59**]. The crystal structure of a related sulfonamide-containing compound PLX3203 bound to BRAF-V600E mutant (pdb id 4FK3) [**59**] has revealed a structural arrangement in which the inhibitor binds to the DFG-in/αC-helix-out conformation of the first monomer, while the second monomer is ligand-free and assumes an active DFG-in/αC-helix-in conformation (**Figs 1 and 2**). Vemurafenib is a type I½ BRAF inhibitor that is FDA-approved for the treatment of highly prevalent BRAF-V600E dependent metastatic melanomas [**60**]. However, Vemurafenib can promote dimerization and transactivate drug-free protomers, leading to paradoxical pathway activation and enhanced signaling in the BRAF-WT cells. The crystal structure of BRAF-V600E oncogenic mutant in complex with Vemurafenib (PLX4032) showed that the inhibitor binding to a DFG-in/αC-helix-out kinase conformation of the first monomer induced a drug-free DFG-in/αC-in active conformation of the second monomer (**Figs 1 and 2**) [**60**]. Dabrafenib is another type I½ inhibitor that binds to both monomers with similar DFG-in/αC-helix-out conformations (pdb id 4XV2, 5CSW) (**Figs 1 and 2**) [**61, 62**]. Dimer-promoting potential of these BRAF inhibitors may be associated with the extent of paradoxical activation. Indeed, while Dabrafenib is a relatively strong inducer of dimerization and paradoxical activation, Vemurafenib may cause a weaker dimer formation and a reduced paradoxical activation in biological assays [**60–62**].

The recent discovery of BRAF inhibitors that can evade paradoxical activation has shown that small changes in the type I½ inhibitors may produce markedly different functional effects by disrupting kinase activation and inhibiting signaling in the BRAF-ERK-MAPK pathways [**63**]. The prime examples of these inhibitors are PLX7904 and its optimized analogue PLX8394 that are very similar to Vemurafenib in their chemical structure. These inhibitors selectively bind and inhibit the activity of both BRAF-WT and mutated BRAF forms, showing efficacy against tumors that are resistant to other BRAF agents. The crystal structures of the BRAF-V600E complex with PLX7904 (pdb id 4XV1), the BRAF-V600E mutant bound with PLX7922 (pdb id 4XV3), and the BRAF-PLX5568 complex (pdb id 4XV9) have revealed a binding mode and interaction profiles that are highly reminiscent of the BRAF-Vemurafenib complex. A weak paradox inducer Vemurafenib and a paradox breaker PLX7904 bind to a similar DFG-in/αC-helix-out kinase conformation in the first monomer, while inducing a drug-free DFG-in/αC-in active conformation of the second monomer (**Figs 1 and 2**). By combining structure-functional analysis and computational modeling, a recent study has argued that small chemical and structural differences between paradox inducers and paradox breakers may produce appreciable changes in the collective dynamics of the BRAF dimer complexes [**64**]. According to this study, paradox inducers may enhance concerted movements of the αC-helix and A-loop regions, while paradox breakers could restrict the αC-helix dynamics and promote more flexible inter-lobe motions. Collectively, structural and functional studies have demonstrated that different classes of BRAF inhibitors can uniquely exploit the mechanism of BRAF activation and act as allosteric modulators of dimerization-induced regulation.

While many computational investigations have been conducted to elucidate molecular mechanisms of the ErbB kinases [**65–71**], molecular modeling and simulation studies of BRAF kinase dynamics, binding and regulation have been fairly scarce [**72–77**]. Most of these studies have analyzed conformational dynamics of the BRAF kinase catalytic domain and have not thoroughly addressed the molecular basis of ligand-induced BRAF dimerization and paradoxical activation. Molecular mechanisms underlying the effects of inactivating BRAF mutations were explored in molecular dynamics (MD) simulations, showing that that the loss of BRAF function upon G596R alteration may depend on cooperative structural changes near the catalytic residue D594 of the DFG motif [**72**]. A combination of docking, MD simulations and QM/MM calculations was used to quantify binding and activity of type I BRAF inhibitors [**73**]. MD simulations and binding free energy calculations have been employed in computational studies of type II DFG-out inhibitors in complexes with the BRAF-V600E mutant [**75**]. Enhanced MD simulations have suggested a mechanism in which oncogenic BRAF mutations could kinetically trap the kinase in the active form by increasing the barriers of conformational transitions between the inactive and active kinase states [**76**]. By combining MD simulations and protein structure network analysis, we have recently examined binding of BRAF dimers with several type I and type II inhibitors [**77**]. Despite the growing effort to understand mechanisms of BRAF regulation, there is a significant gap between detailed structural information about the BRAF dimer complexes and limited biophysical and dynamic characterization of the dimer-promoting potential of BRAF inhibitors and its linkage to paradoxical activation.

In the current study, we expanded our previous work [**77**] by systematically exploring molecular mechanisms of ligand-induced BRAF dimerization and paradoxical activation by a representative panel of type I½ inhibitors. The primary focus of this work was to identify molecular drivers that could differentiate paradox inducers PLX4720, Vemurafenib and Dabrafenib from a new generation of paradox breaker inhibitors exemplified by PLX7904. We characterized differences in the conformational dynamics and collective motions of the BRAF dimer complexes to show that paradox breaker may enforce structural and dynamic preferences of the inactive BRAF-WT monomer, which may be required to escape paradoxical activation. Allosteric regulation of multimeric protein complexes is often associated with ligand-induced cooperative effects. While positive cooperativity is a common mechanism for increasing the binding potential [**78, 79**], negative cooperativity occurs when ligand binding to the first binding site decreases the binding affinity in a second binding site, which is often accompanied by conformational entropy changes [**80–82**]. We reported the binding free energy analysis of the BRAF dimer complexes revealing that negative cooperativity and dimer-promoting potential of the type I½ inhibitors may be important molecular determinants of paradoxical activation. The relationships between inhibitor binding, protein dynamics and allosteric signaling in the BRAF dimers were also explored using a network-centric approach. A graph-based representation of protein structures was used as a framework for structural analysis of the residue interaction networks [**83–85**], identification of functional residues [**86]** and modeling of allosteric communications [**87**]. To characterize functional sites of allosteric regulation, we also explored coevolutionary relationships between BRAF residues. According to statistical coupling analysis (SCA) [**88–90**] and mutual information (MI) model [**91–95**] functional residues in protein systems are often coevolutionary connected. Networks of residues with high mutual information can characterize structural proximity of functionally important sites and this evolutionary signature can distinguish conserved functional residues [**92–95**]. Moreover, coevolving residues are often located close to each other in the protein structure [**96,97**] and may form independent structural modules (or protein sectors) with distinct biochemical functions [**88–90,98,99**]. Functional conformational changes could be often enabled through networks of evolutionarily coupled residues [**100**].

In the current work, we introduce a generalized network model of protein structure and dynamics in which coevolutionary residue dependencies and dynamic maps of residue correlations are integrated in the construction of the residue interaction networks. Different metrics that characterize topology of these networks were compared to understand mechanisms by which BRAF inhibitors selectively modulate the distribution of global mediating centers and allosteric signaling. We show that dimer-promoting inhibitor potential may be coupled with the induction of paradoxical activation through dynamic changes in the residue interaction networks controlled by the αC-helix and dimer interface sites. The results of this study suggest that molecular determinants underlying unique effects of paradox inducers and breakers may be associated with the differences in robustness and efficiency of allosteric interaction networks in the BRAF complexes. By simulating ensembles of allosteric communication pathways in the BRAF dimer complexes, we establish that paradox inducer and breaker inhibitors may activate specific signaling routes that correlate with the extent of paradoxical activation.

## Results and Discussion

### Atomistic and Coarse-Grained Simulations of the BRAF Dimers Reveal Ligand-Induced Modulation of Collective Dynamics

**Fig 3:**
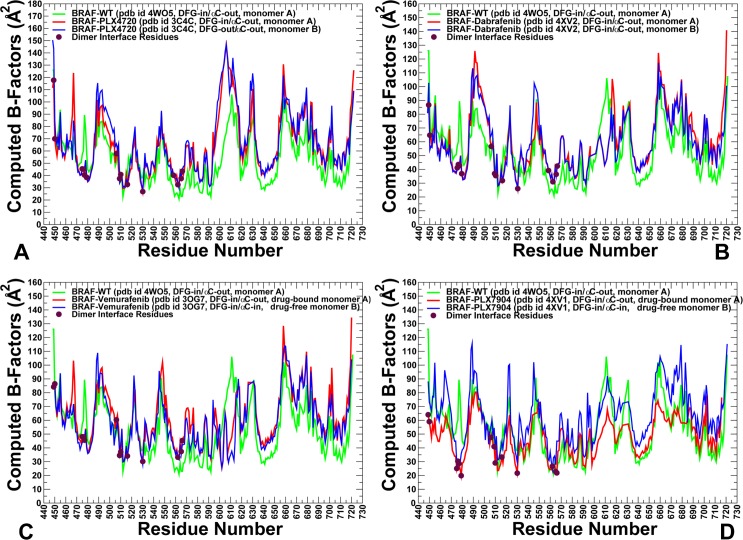
Conformational Dynamics Profiles of the BRAF Structures. Conformational dynamics profiles of the BRAF dimer complexes with PLX4720 (pdb id 3C4C) (A), Dabrafenib (pdb id 4XV2) (B), Vemurafenib (pdb id 3OG7) (C), and PLX7904 (pdb id 4XV1) (D). Conformational mobility profiles of the BRAF dimer complexes are plotted with the reference to the dynamics profile of the BRAF-WT monomeric structure (pdb id 4WO5). The computed B-factors are annotated and colored as follows: the BRAF-WT monomer (in green), the first monomer of the BRAF dimer (in red) and the second monomer of the BRAF dimer (in blue). The positions of the dimer interface residues (D449, W450, K475, W476, H477, D479, R506, R509, H510, F516, Q562, D565, and Y566) are shown on the dynamics profiles filled maroon circles.

All-atom MD simulations of the BRAF dimers in complexes with a panel of type I½ inhibitors were carried out to determine whether conformational dynamics of the BRAF structures would differentiate between paradox inducer and breaker inhibitors. To characterize the inhibitor-induced changes, we compared conformational dynamics profiles of the BRAF dimer complexes with respect to the inactive BRAF-WT monomer (**Fig 3**). We particularly examined conformational variations of the regulatory motifs that are responsible for large conformational transformations in the kinase structures: αC-helix (residues 491–505), αC-β3-loop (residues 485–490), αC-β4-loop (residues 506–516), and the A-loop (residues 593–616). Although the inactive kinase states are generally flexible and could exhibit dynamic inter-lobe movements coupled with positional changes of the αC-helix, the monomeric structure of the inactive BRAF-WT is stabilized in its dormant state through additional hydrophobic interactions between the αC-helix-out and a short helical segment of the A-loop [**38,39**]. We found that binding of paradox inducers may result in the increased mobility of the kinase domain in the BRAF dimer complexes (**Fig 3A–3C**). Moreover, conformational variations in the second monomer were typically greater in the BRAF dimer structures. In particular, thermal fluctuations of the αC-helix and the adjacent αC-β3-loop were markedly larger in the BRAF dimer structures as compared to the inactive BRAF-WT monomer. As a result of these dynamic changes, paradox inducers may promote αC-helix 'in–out' oscillations in the second monomer, leading to the increased population of the closed states with a properly aligned dimer interface. By elevating flexibility of the kinase domain, type I½ paradox inducers may release the constraints of the inactive monomeric form and promote dimerization, which could offset their inhibitory effects. Instructively, binding of paradox breaker PLX7904 seemed to incur dynamic changes that enforce structural preferences of the inactive BRAF-WT monomer in the drug-bound monomer, while increasing conformational mobility of the drug-free monomer (**Fig 3D**). In particular, partly suppressed fluctuations of the αC-β3-loop and the αC-helix in the drug-bound conformation were comparable to the ones seen in the inactive BRAF-WT monomer. Accordingly, PLX7904 binding may restrain flexibility of the αC-β3-loop and encourage the αC-helix region to maintain its nonproductive ‘out’ position, therefore closely mimicking structural environment of the inactive BRAF-WT monomer. At the same time, the concomitantly increased mobility of the drug-free monomer could compromise cooperative interactions between the two monomers and weaken the dimerization interface. According to the recent studies, modifications in the αC-β3-loop that reduce its length could modulate conformational preferences of the αC-helix causing resistance to type I½ inhibitors [**101,102**]. Our findings corroborate with these experiments, suggesting that the dimer-promoting potential of paradox inducers may be linked with cooperative structural shifts of the αC-helix.

We also analyzed how inhibitor binding may affect conformational dynamics of the key dimer interface residues (D449, W450, K475, W476, H477, D479, R506, R509, H510, F516, Q562, D565, and Y566). The dimer interface is formed by several spatially separated interaction sections of the catalytic core. In our simulations, these regions showed moderate thermal fluctuations consistent with their role in modulating stability of the dimer interface. Of particular interest was the dynamics of the central interface section that includes residues D449, W450, R506, R509, and F516. The regulatory residues from the αC-β4 region (R509 and F516) are stable in both monomer and dimers structures and correspond to the hinge sites that coordinate positional shifts of the αC-helix (**Fig 3**). However, the interface residues from the N-terminal tail region (D449, W450) could enjoy a considerable flexibility in the unliganded monomer but become rigidified upon dimer formation. Interestingly, some residues from the peripheral interface section (H577, R558, R562, D565, and Y566) may be also stable in the monomeric form and structurally preorganized for productive inter-monomer contacts in the dimers. Hence, spatially separated interface sections may be predisposed for dimerization, suggesting that allosteric interactions may be transmitted via of diverse communication routes. Based on these findings, we hypothesize that paradox inducer and breakers could selectively modulate stability of the dimerization interface regions and activate specific signaling pathways. To summarize, despite a similar pattern of the conformational fluctuation profiles in the BRAF dimer complexes, we detected specific dynamic signatures that could differentiate paradox inducer and paradox breaker inhibitors. The central finding of this analysis was that paradox breaker PLX7904 in its drug-bound conformation could strictly impose structural and dynamic features of the inactive BRAF-WT monomer that may be required for evading paradoxical activation.

Atomistic simulations suggested that the type I½ inhibitors may induce changes in the collective dynamics of the BRAF dimer structures that may be associated with their dimer-promoting abilities and paradoxical activation. To characterize collectivity of protein motions in the BRAF dimers, we complemented all-atom MD simulations with elastic network modeling (ENM) that can describe large cooperative transformations of protein regions in the space of slow low-frequency modes. According to this model, salient properties of protein dynamics can be uniquely defined by structural architecture of the protein fold and ligand-induced conformational changes may be embedded in the native fluctuations of the unbound proteins [**103**]. The essential mobility profiles of the BRAF structures were obtained using the Gaussian network model (GNM) [**103–105**]. In this model, the distribution peaks point to the protein regions involved in large structural movements, while local minima correspond to the functional residues that remain immobilized and serve as global hinge centers that coordinate collective dynamics. We first analyzed collective dynamics of the BRAF-WT monomeric state in the space of the slowest low-frequency modes (**S1 Fig**). The first principal mode of the BRAF-WT kinase domain corresponded to coordinated rotations of the N-terminal lobe and C-terminal lobe in opposite directions. In this mode, the distance fluctuations between residues within each lobe are small and residue movements are mostly positively correlated, while the inter-residue distance deviations between lobes are large as motions of these residues are negatively correlated (**S1 Fig**). In the second slow mode, the kinase lobes undergo coordinated opening and closing movements with respect to each other, which may be important for ATP and ligand binding. In the third principal mode, the kinase lobes undergo a shear motion sliding along their interface section which is accompanied by the forward move of one lobe and a concomitant inward displacement of the other lobe. We argue that the conservation of major slow modes in the kinase domain may be required for induction of functional motions during diverse regulatory processes. These principal motions of the catalytic domain are conserved across the protein kinase family as was affirmed in the NMR experiments [**106,107**] and reproduced in computational studies [**108,109**].

**Fig 4:**
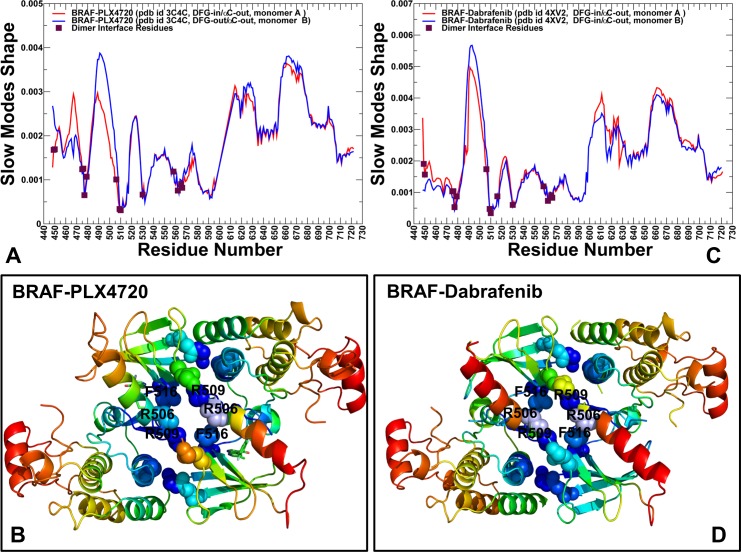
The GNM-Based Essential Mobility Profiles of the BRAF Dimer Complexes with PLX4720 and Dabrafenib. Collective dynamics of the BRAF dimers is analyzed using the cumulative contributions of the first five slowest GNM modes. Conformational mobility profiles and structural maps of collective motions are shown for the BRAF dimer complex with PLX4720 (pdb id 3C4C) (A, B) and Dabrafenib (pdb id 4XV2) (C, D). The slow mode shapes are annotated and colored in red (first monomer) and blue (second monomer). Structural maps of collective dynamics are based on fluctuations driven by the slowest five modes. The color gradient from red to blue indicates the increasing structural rigidity and refers to an average value over the backbone atoms in each residue.

**Fig 5:**
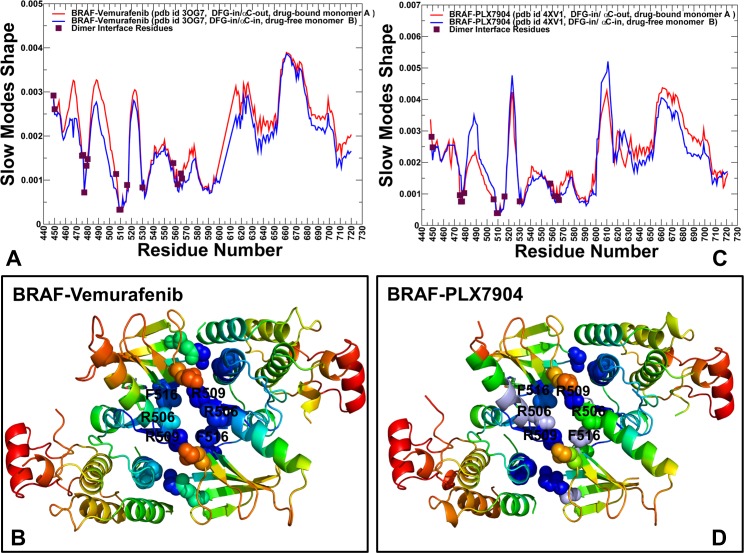
The GNM-Based Essential Mobility Profiles of the BRAF Dimer Complexes with Vemurafenib and PLX7904. Collective dynamics of the BRAF dimers is analyzed using the cumulative contributions of the first five slowest GNM modes. Conformational mobility profiles and structural maps of collective motions are shown for the BRAF dimer complex with Vemurafenib (pdb id 3OG7) (A,B) and PLX7904 (pdb id 4XV1) (C,D). The slow mode shapes are annotated and colored in red (first monomer) and blue (second monomer). Structural maps of collective dynamics are based on fluctuations driven by the slowest five modes.

Functional motions along the slowest modes showed interesting differences between the BRAF dimer structures (**S2 Fig**). The principal movements of the first and second monomers were similar in the BRAF dimers complexed with paradox inducers PLX4720 and Dabrafenib. However, in the BRAF complexes with Vemurafenib and PLX7904, collective movements of the second monomer were reduced near the αC-β3-loop (residues 485–490) and secondary interface section (residues H510, K475, W476, H577, R558, R562, D565, and Y566) (**S2 Fig**). Global motions around this hinge site may compromise effective dimerization and reduce the extent of paradoxical activation. By examining collectivity of the slow modes in the BRAF dimer structures we found that up to five low-frequency modes may be required to adequately describe all global movements (**S3 Fig**). Subsequent slow modes have a markedly reduced level of collectivity and could only affect local dynamics changes (**S3 Fig**). The degree to which these slow GNM modes capture global structural changes was estimated by the cumulative overlap with the principal component modes derived from conformational variability in the BRAF dimer crystal structures [**110,111**]. The slowest five modes exhibited more than 90% overlap with the principal components of structural changes observed in the BRAF dimer complexes with different classes of inhibitors **(S3 Fig).** Based on the observed convergence of slow modes, we analyzed the essential mobility profiles from the cumulative contribution of the first five principal modes and computed the mean square displacements of protein residues driven by these slow modes. We observed that dimer formation may introduce an additional cooperative mode with an extended global hinge that runs across the entire inter-monomer interface (**Figs 4 and 5**). This slow mode forms a dominant direction of collective dynamics in the BRAF dimers, where the rigid interfacial sites become colocalized with the αC-helix residues. The analysis of collective dynamics in the BRAF complexes with paradox inducers PLX4720 and Dabrafenib (**Fig 4**) showed a dominant peak corresponding to a highly flexible αC-β3-loop (residues 485–490) that controls structural preferences of the αC-helix. In these complexes, the central interface section forms a core of the extended hinge region that could promote functional movements of the αC-helix around the crystallographic inactive ‘out’ position. At the same time, the key residues in the secondary sections of the dimer interface (H510, K475, W476, H577, R558, R562, D565, and Y566) were more flexible. As a result, the collective movements of these BRAF dimers may involve concerted changes of the αC-helices around the central hinge and allow for proper alignment of the hydrophobic spines and the dimer interface. In the BRAF-PLX4720 complex, the second monomer adopts a more flexible DFG-out/αC-helix-out conformation, leading to larger movements in the αC-β3-loop (residues 485–490) and αC-helix (residues 491–505) (**Fig 4A and 4B**). A similar pattern of collective motions could be seen the BRAF-Dabrafenib complex (**Fig 4C and 4D**). Collective dynamics profiles in the BRAF complexes with Vemurafenib (**Fig 5A and 5B)** and PLX7904 (**Fig 5C and 5D**) revealed that the paradox breaker could suppress movements of the αC-helix region, thereby compromising the proper alignment of the regulatory residues and interfacial residues in the global hinge. On the other hand, the ‘peripheral’ sections of the interface become more immobile (**Fig 5C and 5D**). Hence, though the overall shape of the extended hinge may be preserved in all BRAF dimer structures, paradox breaker could engineer changes in several key residues that would shift the major hinge center away from the central interface section. The central finding of this analysis was that paradox breaker may enforce global dynamic preferences of the inactive BRAF-WT monomer that would likely compromise coordinated allosteric changes in the αC-helix and dimer interface regions. This dynamic effect of PLX7904 may intervene with the effective side-to-side dimerization and therefore alleviate paradoxical activation.

Interestingly, a number of dimer interfacial residues (R509, H510, H577, R558, R562, D565, and Y566) are structurally rigid along slow modes in the BRAF-WT monomer. In particular, the side-to-side dimer architecture and central interface section are anchored by a critical residue R509 that is a rigid hinge site in the inactive monomeric structure (**Figs 4 and 5**). Hence, structural stability of these sites may be determined by their global dynamics rather than local stabilization in a particular kinase conformation. These findings are consistent with the notion that global hinge centers that control collective dynamics may be intrinsically determined by the native protein topology and retain their mechanical role upon dimer formation and binding with inhibitors [**112–114**]. Differences in collective dynamics of the dimer structures may be associated with ligand-induced changes in the essential mobility of the αC-helix residues R506 and F516. These residues couple the αC-helix motions with the dimer interface interactions and are likely to be critical for the mechanism of paradoxical activation. Our results indicated that paradox inducer and paradox breaker inhibitors could selectively alter the essential mobility profile of several important sites in the global hinge region, while preserving the general shape of the slow modes and collective motions.

### Negative Cooperativity and Dimer-Inducing Potential of the BRAF Inhibitors Are Drivers of Paradoxical Activation

**Fig 6:**
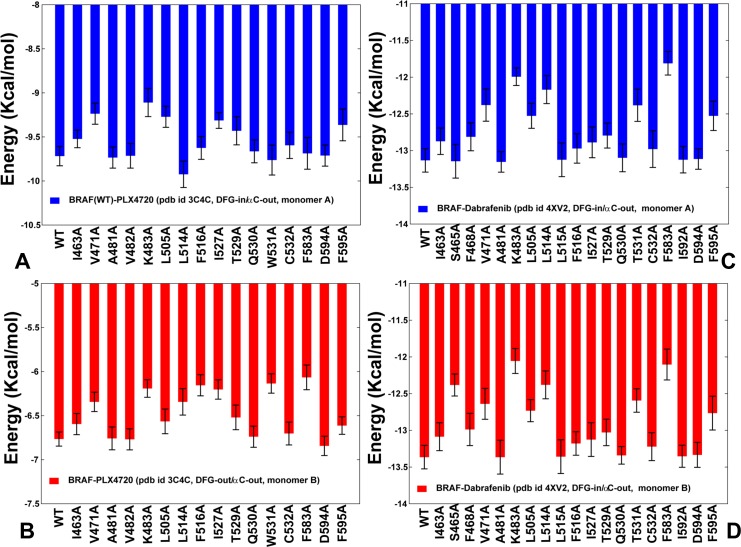
Binding Free Energy Calculations of the BRAF Dimer Complexes with PLX4720 and Dabrafenib. Binding free energies and alanine scanning of the binding site residues in the first and second binding sites of the PLX4720-BRAF complex (A,B) and Dabrafenib-BRAF complex (C,D). The standard errors of binding free energy differences, which are the standard deviation of the mean values, were ~ 0.11–0.25 kcal/mol for the PLX4720-BRAF complex and 0.18–0.22 kcal/mol for the Dabrafenib-BRAF complex. The results of alanine scanning are shown in blue bars for the first binding site (A, C) and in red bars for the second binding site (B, D).

**Fig 7:**
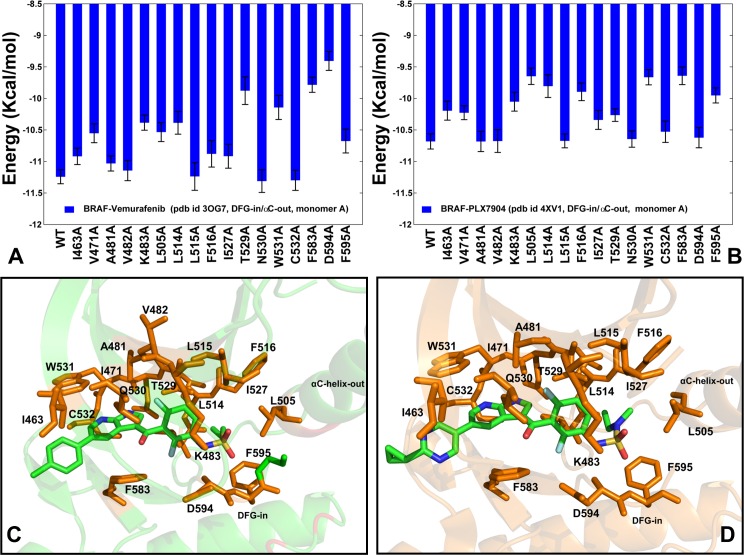
Binding Free Energy Calculations of the BRAF Dimer Complexes with Vemurafenib and PLX7904. Binding free energies and alanine scanning of the binding site residues in the drug-bound monomer of the Vemurafenib-BRAF complex (A) and the PLX7904-BRAF complex (B). The standard errors of binding free energy differences, which are the standard deviation of the mean values, were ~ 0.15–0.25 kcal/mol for the Vemurafenib-BRAF complex and 0.2–0.3 kcal/mol for the PLX7904-BRAF complex. A close-up of the Vemurafenib binding mode (C) and PLX7904 binding mode (D). The inhibitor is shown in atom-colored sticks and annotated. The binding site residues are shown in orange sticks and annotated.

Structural analysis of the BRAF dimer complexes showed a considerable similarity of the inhibitor binding modes in the first monomer, while binding of PLX4720 and Dabrafenib to the second binding site produced a different interaction pattern (**Fig 2**). To identify the binding energy hotspots and quantify binding energetics in the first and secondary binding sites, we performed MM-GBSA calculations **[115,116**] of the BRAF dimer complexes. In these simulations, we examined the molecular basis of negative cooperativity that may be induced by the BRAF inhibitors. MM-GBSA calculations of the BRAF dimer complexes were supplemented by a systematic alanine scanning of the binding site residues [**117,118**] for each of the studied complexes. Our results reproduced the relative order of binding for the inhibitors (**Figs 6 and 7**), particularly demonstrating a stronger binding affinity of Dabrafenib as compared to Vemurafenib and PLX4720. According to the experimental data, Dabrafenib binds to the BRAF kinase with IC50 ~6 nM [**54**] and PLX4720 binds BRAF with IC50 = 160 nM [**59**], while Vemurafenib showed weaker binding with IC50~260–360 nM [**54, 60**].

Computational alanine scanning identified common binding energy hotspots of all studied type I½ inhibitors: V471, V482, K483, L505, L514, F516, W531, F583, and F595 residues (**Figs 6 and 7**). Some of these binding hotspots are located in the regulatory regions and selectivity pocket that are involved in collective dynamics of the BRAF dimers and allosterically coupled with the dimer interface residues. In our previous work we demonstrated that type I BRAF inhibitors may be less sensitive to conformational and variations of the R-spine residues, while more selective type II inhibitors could be strongly coupled with the dynamics of the hydrophobic spines [**77**]. According to alanine scanning performed in the current study, binding of type I½ inhibitors may be strongly influenced by the energetic hotspots from the catalytic salt bridge (K483), the αC-helix (L505), the αC-β4-loop (L514, 516), and the DFG motif (D594, F595). (**Figs 6 and 7**). Some of these sites (L505, L514, F516) were especially critical for binding of paradox breaker PLX7904 as alanine modifications of these residues caused a substantial reduction in the binding affinity (**Fig 7B and 7D**). These residues link the αC-helix movements with the central section of the dimer interface (R506, R509, F516, and W450) and may be important for the inhibitor-induced dimerization and activation. Our results are consistent with mutagenesis studies that affirmed the critical role of these residues for the inhibitor binding [**119,120**]. According to these experiments, L505H substitution is a spontaneously occurring oncogenic BRAF mutation that can confer resistance to PLX4032 and Vemurafenib. Moreover, BRAF-L505H mutant can activate MAPK signaling, but has a smaller activating and oncogenic potential than the prevalent BRAF-V600E mutation. Paradox breaker PLX7904 is more sensitive to BRAF-L505H than PLX4720, but still insufficiently effective to overcome drug resistance conferred by this mutant [**120**]. Notably, only a new paradox breaker inhibitor PLX8394 can successfully combat mechanisms of drug resistance and inhibit the MAPK pathway in the BRAF mutant cells that are resistant to Vemurafenib and PLX7904 [**120**].

We also computed binding affinities and carried out alanine scanning of PLX4720 and Dabrafenib interactions in the second binding site (**Fig 6B and 6D**). These results revealed a drastically reduced binding affinity of PLX4720 in the secondary site, where the inhibitor assumed a different binding mode and was bound to the DFG-out/αC-helix-out conformation (**Fig 6B**). As a result, even though PLX4720 occupies both monomers in the crystal structure, it may effectively inhibit only the first monomer. The observed negative cooperativity of PLX4720 may be associated with the increased conformational mobility of the second monomer, which is consistent with the reduced occupancy and faster off-rates of PLX4720 dissociation seen in the experiments [**59**]. The observed connection between the reduced inhibitor binding and the increased flexibility in the secondary monomer reflects the salient feature of negative cooperativity. Indeed, this effect is associated with allosteric mechanisms that occur without global structural changes but could trigger significant changes in conformational entropy [**121,122**]. Our results corroborated with the experimental studies showing that binding of some BRAF inhibitors may be often accompanied by negative cooperativity effects that promote paradoxical activation [**123**]. Dabrafenib binding induced similar structural environment of the binding sites and concerted motions of the two monomers, leading to small differences in the binding free energies between the first and second monomers (**Fig 6B and 6D**). Accordingly, this inhibitor may display an appreciable dimer-promoting potential but only a marginal negative cooperativity. Hence, dimerization and the induction of paradoxical activation by type I½ inhibitors may be associated with a different degree of negative cooperativity. Our results indicated that dimer-promoting potential and negative cooperativity of type I½ inhibitors may represent important molecular determinants of BRAF regulation and paradoxical activation.

### Protein Sectors of Coevolutionary Residues Mediate Dimerization in the BRAF Complexes

**Fig 8:**
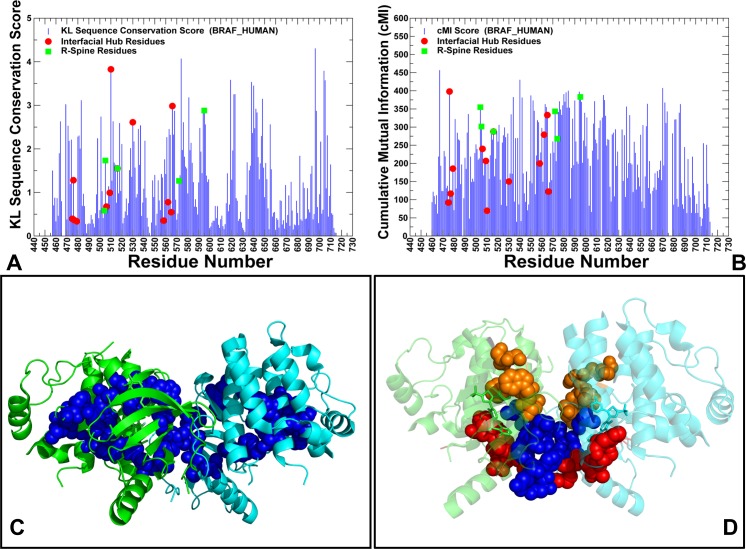
Coevolutionary Dependencies of the BRAF Kinase Residues. Sequence conservation and coevolutionary propensities of the BRAF residues. (A) The KL conservation score. The residue numbering in the sequence conservation profile corresponds to the residue numbering in the BRAF crystal structures. (B) The cMI profile measures cumulative accumulation of mutual information per residue. The residue profiles are shown in blue bars. The inter-domain interface residues are shown in red circles. The R-spine residues are highlighted in green squares. (C) Structural mapping of high cMI residues (in blue spheres) onto the crystal structure of the Vemurafenib-BRAF complex (pdb id 3OG7). These residues included V471, A481, L515, F516, W531, L537, H574, L577, K578, S579, F583, L584, F595, W619, M620, D638, Y640, F642, and I644. The first monomer is shown in green ribbons and the second monomer is shown in cyan ribbons. (D) Structural mapping of the protein sectors of coevolving residues is shown in both monomers. The binding site sector residues Q530, W531, C532, F583, and S536 are shown in orange spheres; the regulatory sector residues R506, F516, I572, H574, H568, and F595 are shown in red spheres and the dimerization sector residues D449, W450, E451, L505, R509, L514, and F516 are shown in blue spheres.

Conformational dynamics and binding energetics of the BRAF dimer complexes suggested that paradoxical activation may be associated with ligand-induced modulation of allosteric interactions. To investigate this relationship, we proposed a model that incorporated both dynamic and coevolutionary residue correlations in the construction and analysis of the residue interaction networks. First, we explored coevolutionary residue dependencies to test whether key functional sites mediating allosteric interactions in the BRAF dimer complexes may display a strong coevolutionary signal. By using MI approach [**92–95**] we evaluated the Kullback-Leibler conservation score (**Fig 8A**) and the cumulative mutual information (cMI) score that estimates the degree of shared MI of a given residue with other protein residues (**Fig 8B**). Highly coevolving residues were primarily assembled in the ATP binding site, in the catalytic loop region and near the substrate binding motif (residues 619-WMAPE-623). Structural mapping of the high cMI residues revealed a well-connected network with a characteristic θ-like shape [**99,124**] in which coevolutionary networks connected the ATP binding site with the substrate binding region through the hydrophobic spines (**Fig 8C**). In this network, the dimer interface residues R506 and R509 may be directly coupled with the R-spine residues L505 an F516. Hence, functional residues involved in collective motions and dimerization may be also coevolutionary coupled. We also found that coevolutionary residues may form three independent structural sectors: the binding site sector (Q530, W531, C532, F583, S536), the regulatory region sector (R506,F516, I572, H574, H568, F595) and the dimerization sector (D449, W450, E451, L505, R509, L514) (**Fig 8D**).

These sectors of coevolving residues comprise contiguous clusters that only partially overlap and involve key residues from the ATP-binding site and regulatory αC-helix and αC-β4-loop regions. The topology and functional role of these modules are consistent with the main characteristics of protein sectors such as physical connectivity in the tertiary structure and biochemical independence in mediating protein function [**88–90**]. Strikingly, several key residues (L505, R509, L515, and F516) are strategically located at the cross-section of coevolving modules connecting the ATP binding site with the regulatory αC-helix and the dimer interface. These observations suggested that key regulatory residues connecting the αC-helix and the dimer interface may be subjected to coevolutionary constraints. Structural mapping of protein sectors also indicated that these modules of highly coevolving BRAF residues comprise a global interaction network that may promote allosteric interactions and facilitate conformational changes during dimerization.

**Fig 9:**
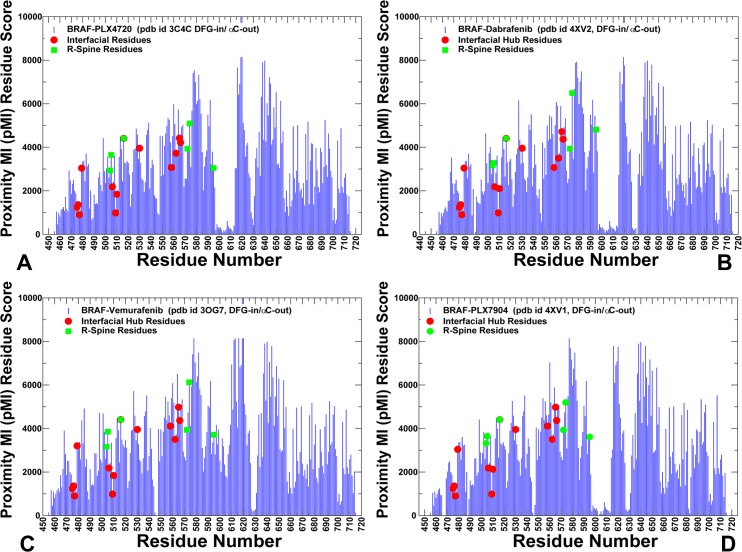
Proximity-based Coevolutionary Profiles of the BRAF Dimer Complexes. The ensemble-based pMI profiles of the BR AF complexes with PLX4720 (A), Dabrafenib (B), Vemurafenib (C), and PLX7904 (D). pMI values for each residue position are evaluated as the sum of cMI values of all residues within 5Å distance from a given residue. The distance between each pair of residues in the structure was calculated as the shortest distance between any two non-hydrogen atoms from respective two residues. pMI profiles are computed using average values obtained from MD trajectories and ensemble-based definition of the local residue environment. The residue profiles are shown in blue bars. The inter-domain interface residues are shown in red circles. The R-spine residues are highlighted in green squares.

We also computed proximity-based mutual information (pMI) score (**Fig 9**), which is a structure-sensitive parameter defined as the average of cMI scores of all residues within a predefined distance from a given residue. Importantly, many regulatory sites displayed high pMI scores, suggesting that to maintain a particular function their local structural environment may be enriched by highly coevolving residues (**Fig 9**). Interestingly, the dimer interface residues are located in structural proximity of high pMI sites. One group of the interfacial residues (D449, W450, R506, and R509) is linked to the high pMI residue F516. Another interfacial cluster (R558, R562, D565, and Y566) is located near the R-spine residues I572 and H574 that also showed high pMI score (**Fig 9**). We argue that functional importance of the R-spine residues as drivers of regulatory changes may be determined by their coupling to clusters of flexible coevolving residues that undergo coordinated conformational changes upon dimerization and activation. This may highlight the functional role of the interfacial residues as transmitters of allosteric signals and conformational changes that are orchestrated by the regulatory spine residues.

### BRAF Inhibitors Modulate Centrality and Communication Propensity of Allosteric Centers in the Residue Interaction Networks

**Fig 10:**
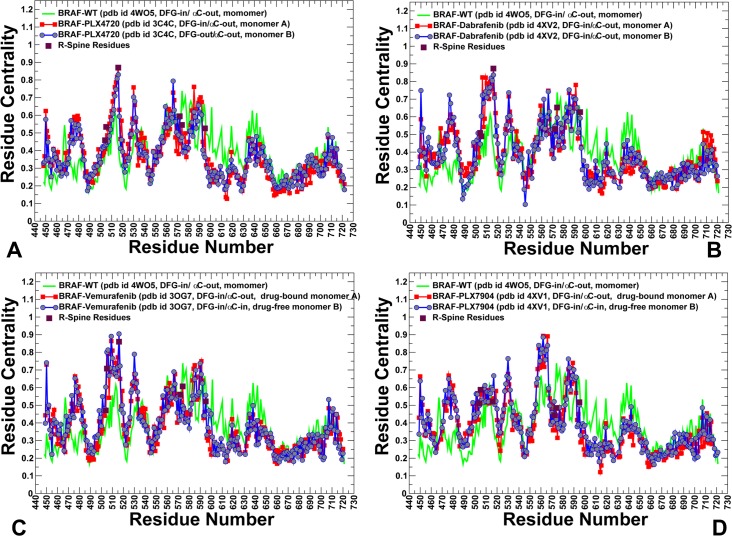
Residue-Based Centrality Profiles in the BRAF Structures. The residue centrality profiles of the BRAF dimer complexes with PLX4720 (pdb id 3C4C) (A), Dabrafenib (pdb id 4XV2) (B), Vemurafenib (pdb id 3OG7) (C), and PLX7904 (pdb id 4XV1) (D). The distribution for the BRAF-WT (in green) is shown for comparison on all panels. The centrality profiles for the first monomer are shown in red lines with filled red squares and for the second monomer in blue lines with filled blue circles. The centralities of the R-spine residues in the drug-bound monomer are highlighted as filled maroon squares.

A novel methodological aspect of this work was an integration of coevolutionary residue dependencies and dynamic maps of residue correlations in the construction and analysis of the residue interaction networks. In this model, residues with high level of centrality correspond to key mediating centers that connect coevolutionary and dynamically coupled residues in a global interaction network. By employing this protein structure network model, we determined how paradox inducer and paradox breaker inhibitors could modulate organization of the interaction networks and activation pathways. Different network parameters such as residue-based centrality and communication propensity were explored to characterize how BRAF inhibitors could affect mediating capabilities of kinase residues and the distribution of allosteric centers. First, we compared the residue centrality profiles of the BRAF-WT monomer structure and BRAF dimer complexes (**Fig 10**). The residue centrality of the BRAF-WT monomer revealed several conserved peaks corresponding to the αC-helix region (residues 491–516), the 574-HRD-576 catalytic motif, the 593-DFG-595 motif, and the organizing αF-helix (residue 635–651). The integrating role of the αC-helix and the αF-helix regions is known to be critical for modulation of kinase dynamics and activity [**5–10, 65**]. According to our results, paradox inducer inhibitors could alter centrality of the kinase residues by amplifying the peak near the αC-helix/αC-β4-loop region and central section of the dimer interface (R506, R509, F516, and W450) (**Fig 10A–10C**). In some contrast, paradox breaker PLX7904 may induce important network changes by reducing centrality of the regulatory αC-helix region to the level observed in the BRAF-WT monomer (**Fig 10D**). The major peak of the distribution shifted towards the secondary interfacial cluster formed by C-terminal residues (R558, R562, D565, and Y566). As a result, network preferences of functional centers may be partly distorted in the PLX7904-BRAF complex due to stabilization of a nonproductive αC-helix-out conformation. The impaired allosteric coupling between the αC-helix and the central section of the dimer interface may force the system to “dispatch allosteric traffic” through longer and arguably less efficient paths proceeding through the secondary interfacial cluster. These findings are consistent with the analysis of collective dynamics, showing that paradox breaker may induce dynamic and network signatures that are characteristic of the inactive BRAF-WT monomer.

**Fig 11:**
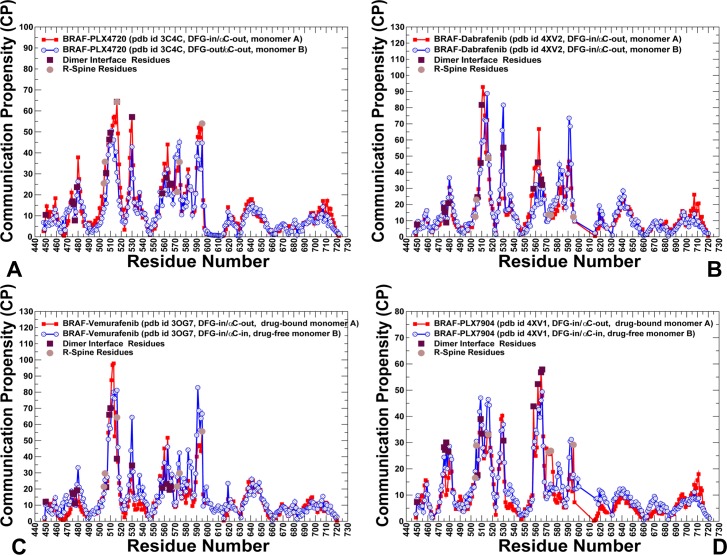
Allosteric Communication Propensities in the BRAF Structures. The residue-based CP profiles of the BRAF complexes with PLX4720 (A), Dabrafenib (B), Vemurafenib (C) and PLX7904 (D). The profiles for the first monomer are shown in red lines with filled red squares and for the second monomer in blue lines with filled blue circles. The R-spine residues are highlighted as filled maroon squares.

By expanding a force constant model of protein dynamics [**125–127**], we computed communication propensities (CP) of protein residues defined as average variations of the effective “distance” metric that measures both distance fluctuations and variations in the pMI scores between a given residue and all other residues. In this model, coevolving and dynamically correlated residues whose effective distances fluctuate with low or moderate intensity are expected to communicate with the higher efficiency than the residues that experience large fluctuations. We found that paradox inducers showed a similar CP profile in which the largest dominant peak corresponded to the αC-helix/ αC-β4-loop region showing high values for the interfacial residues R506, R509, H510, and F516 (**Fig 11A–11C**). In addition to the interfacial residues, the αC-helix and R-spine residues may be also characterized by high communication capabilities. Consequently, by enhancing global mediating capacity of these residues, paradox inducers would likely direct the shortest inter-residue pathways through the central interface section, which could promote the effective side-to-side dimerization and activation. In the BRAF-PLX7904 complex, the CP values of the αC-helix and central interfacial section were markedly lowered (**Fig 11D**). Similarly to the residue centrality profile, the major peak shifted to the secondary interfacial region. Hence, paradox breaker may trigger dislocation of mediating centers in the central hinge region that would weaken dimer formation and favor alternative activation routes. Structural mapping of the interfacial regions illustrated differences in the communication preferences of the interface regions that may affect allosteric signaling in the BRAF dimers (**S4 Fig**). To conclude, our analysis of the residue interactions networks demonstrated that paradox breakers may circumvent paradoxical activation by closely mimicking structural, dynamic and network properties of the inactive BRAF-WT monomer.

### Paradox Inducer and Paradox Breaker Inhibitors Induce Specific Allosteric Communication Pathways

**Fig 12:**
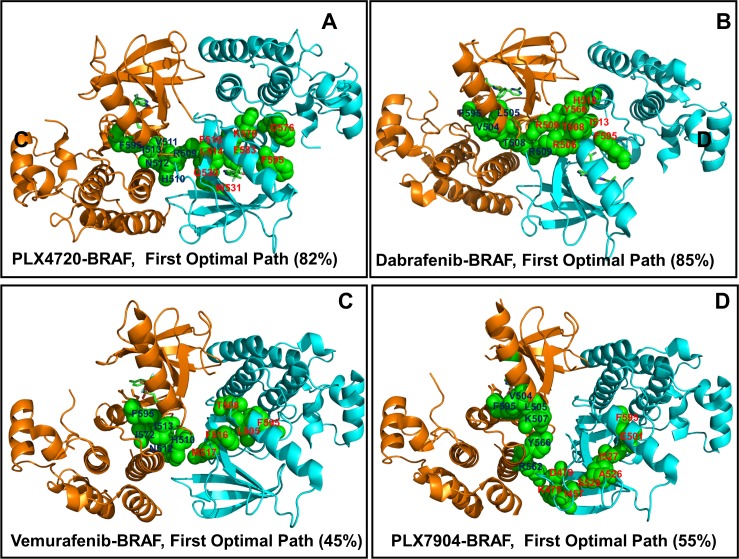
Structural Map of Allosteric Communication Pathways in the BRAF Dimer Complexes. The most probable communication pathways in the PLX4720-BRAF complex (pdb id 3C4C) (A), Dabrafenib-BRAF complex (pdb id 4XV2) (B), Vemurafenib-BRAF complex (pdb id 3OG7) (C), and PLX7904-BRAF complex (pdb id 4XV1) (D). The BRAF structures are shown in ribbons, with the monomer A in orange and monomer B in cyan. The key residues along the communication routes are annotated and shown in green spheres. The error bars on the pathway occupancies are within 5%.

Using matrix of communication distances between protein residues in the BRAF dimer complexes, we obtained ensembles of shortest paths connecting each pair of residue nodes in the BRAF structures. In this model, allosteric communication pathways proceed via a network of coevolving and dynamically coupled residues. By simulating ensembles of short paths that connect the ligand binding sites in the BRAF dimers, we explored whether paradox inducer and breaker inhibitors may activate specific communication pathways and exploit different sections of the dimer interface. We reported atomistic details of the most probable paths that connect the regulatory DFG motifs in the first and second monomers. First, we found that single, high occupancy communication routes may dominate the ensemble in the BRAF dimer complexes with paradox inducers PLX4720 (**Fig 12A**) and Dabrafenib (**Fig 12B**). The most probable path (82% occupancy) in the PLX4720-BRAF dimer connected F595 (monomer A) with I513, V511, and N512 residues from the αC-β4-loop before reaching the key interfacial residue R509 of the monomer A. At this point, the path bridged two monomers by connecting R509 (monomer A) with F516 of the monomer B. The path then navigated through the monomer B, moving from F516 to the binding site residues L514, Q530, W531, C532, and F583 residues (**Fig 12A**). Interestingly, this path traversed through most of the binding energy hotspots in the second monomer. In this BRAF complex, a critical inter-monomer juncture linked R509 (monomer A) with F516 (monomer B) by exploiting the central section of the inter-monomer interface (R506, R509, F516, W450).

Notably, the observed communication pathways navigated through protein sectors formed by the coevolutionary residues. These findings suggested that coevolutionary networks may form direct pathways for transmitting allosteric signals [**100**]. The most probable path in the Dabrafenib-BRAF complex (85% occupancy) could proceed from F595 of monomer A to the R-spine residues of the αC-helix (V504, L505), subsequently reaching to the interfacial residue R509 of the monomer A (**Fig 12B**). At this point, the path bridged over the inter-domain interface by connecting to R506 and R509 of the monomer B. After crossing the dimer interface, the route proceeded to Y566 and H510 residues in the monomer B before reaching I513 of the αC-β4 region and arriving at the final destination F595 of the second monomer (**Fig 12B**). In this case, the most probable path utilized direct coupling of the R-spine residues (V504, L505) with the central interfacial sites R506 and R509. Hence, paradox inducers may facilitate rapid and efficient communication through a single pathway connecting the binding site with the central interfacial section (**S4 Fig)**.

Of special interest was a comparison of communication pathways in the BRAF complexes with Vemurafenib and PLX7904, whose structural and dynamic attributes are very similar. In these structures, signaling pathways become more diverse with 45% occupancy for the most optimal path in the Vemurafenib-BRAF complex and 55% occupancy in the PLX7904-BRAF complex (**Fig 12C and 12D**). The favorable route in the Vemurafenib-BRAF complex proceeded from F595 of the drug-bound monomer to I592 and the αC-β4-loop residues (N512, I513) before reaching H510 residue. At this point, the path crossed the interface to the drug-free monomer, connecting with M517, F516 and the αC-helix L505 residue (**Fig 12C**). The path then linked with T508 before reaching F595 of the second monomer. Interestingly, this communication route proceeded through the interfacial residue H510, while the central section of the interface (R506, R509) was avoided. In the BRAF complex with a paradox breaker PLX7904, the ensemble of communication pathways was also fairly diverse and none of the dominant routes exploited the central interface section. The first optimal path (55% occupancy) was longer and less optimal in comparison with favorable routes found in other BRAF dimers (**Fig 12D**). Starting from F595 residue of the drug-bound monomer, the path preceded to the R-spine residues V504 and L505. The route then followed to K507 before making a turn and reaching out to Y566 and R562 residues. At this point, the path crossed the dimer interface and connected to D479 and K475 residues of the second monomer. Subsequently, this path linked K475 with I457, A526, I527 residues from the N-terminal lobe in the second monomer before reaching to E501 (from the catalytic salt bridge) and connecting to F595 in the second monomer. Hence, the most favorable signaling route in the PLX7904-BRAF complex may bypass the central interface section and navigate through other interfacial residues.

The central finding of this analysis was the emergence of suboptimal and less efficient communication pathways for the paradox breaker as opposed to direct and more efficient transmission routes detected in the BRAF complexes with the paradox inducers.

Rapid and efficient communication in the BRAF complexes with paradox inducers may come at the expense of high dependency on critical modes in the αC-helix, αC-β4-loop, and dimer interface region (R506, R509, and F516). Of particular significance are key residues of the inter-monomer hinge F516 and R509 that control proper alignment of hydrophobic spines and allosteric coupling between the αC-helix and dimer interface. These high centrality residues that minimize the short path length and ensure the efficiency of allosteric communications are experimentally known to be important for kinase activity and regulation. As a result, even minor modifications of these residues could alter activation signaling pathways and trigger drug resistance. A different organization of optimal pathways in the BRAF-PLX7904 complex may reduce the efficiency of allosteric communications and impair activation signaling, while making drug binding more resilient to mutations of allosteric hotspot nodes. In network terms, the increased diversity of communication pathways would likely diminish network efficiency, but may potentially increase level of tolerance against mutations of mediating residues [**128–130**]. By linking paradoxical activation with the ligand-mediated changes in allosteric networks and communication pathways, our results may provide a network-based rationale of the distinct functional effects exerted by the type I½ BRAF inhibitors.

## Conclusions

In this work, we explored molecular mechanisms of dimerization-induced BRAF regulation by a panel of type I½ inhibitors. Our study was motivated by recent revelations that most of the BRAF inhibitors can paradoxically activate kinase by inducing dimerization and promoting dimer-dependent transactivation. These experimental discoveries have shown that therapeutic and clinical utility of BRAF inhibitors that exert paradoxical activation can be diminished, creating a need for a quantitative assessment of dimerization potential induced by the inhibitor. By combining atomistic and coarse-grained simulations with modeling of the residue interaction networks and coevolution, we have identified molecular determinants of differential functional effects produced by a series of paradox inducer and breaker inhibitors. According to our results, dimer-promoting potential and negative cooperativity of type I½ inhibitors may be associated with the induction of paradoxical activation. These functional effects may be controlled by several regulatory sites in the extended hinge formed by the αC-helix and dimer interface residues. The important result of this study is that paradox inducers and paradox breaker may have specific dynamic and network signatures that uniquely characterize their distinct functional effect on BRAF activation. The central finding of our investigation is that paradox breaker PLX7904 may evade activation by closely mimicking structural, dynamic and network properties of the inactive BRAF-WT monomer. We argue that BRAF inhibitors that amplify structural features of the inactive BRAF-WT monomer and suppress dimer-promoting function may alleviate the extent of paradoxical activation and enhance drug efficacy. By simulating allosteric interaction networks and communication pathways in the BRAF structures, we determined that dimerization may promote efficient and robust activation pathways that are enabled through coordinated structural changes of the αC-helix and dimer interface residues. Furthermore, paradox inducer and breaker inhibitors may activate specific signaling routes that correlate with the extent of paradoxical activation. The results suggested that paradox inducer inhibitors may facilitate a rapid and efficient communication by a single pathway, while a paradox breaker may induce a broader ensemble of suboptimal and less efficient communication routes.

Understanding of system-based relationships between network efficiency and drug binding may prove useful in the design of novel BRAF kinase inhibitors. The lessons from our study could also inform discovery efforts aiming in mitigating adverse biological consequences and tailoring existing BRAF inhibitors into paradox breakers. According to the current view, design of type I½ BRAF inhibitors capable of stabilizing a non-productive αC-helix-out conformation may help to block the dimerization interface. Our results suggested that these inhibitor-induced changes may result in the induction of alternative signaling routes and could only partially alleviate dimerization-induced transactivation. An alternative design strategy may involve direct targeting of several binding energy hotspots (L505, R509, L515, and F516) that also serve as critical hubs in the allosteric interaction networks, coevolutionary networks and signaling pathways. By exploiting the unique functional role of these residues and simultaneously enforcing structural features of the inactive BRAF monomer, next generation of sulfonamide inhibitors may have a potential to act as paradox breakers in crystal and cellular environments.

## Materials and Methods

### Atomistic and Coarse-Grained Simulations

MD simulations were performed for the monomeric structure of the BRAF-WT, and BRAF dimer-inhibitor complexes that included BRAF-WT bound with PLX4720 (pdb id 3C4C), BRAF-WT complex with Dabrafenib (pdb id 5CSW), BRAF-V600E bound with Dabrafenib (pdb id 4XV2), BRAF-V600E complexed with Vemurafenib (pdb id 3OG7), and BRAF-V600E complex with PLX7904 (pdb id 4XV1). A combination of several long MD simulations and multiple shorter MD runs was undertaken to ensure a broader and more unbiased sampling. We have carried out two independent 500 ns and five independent 200 ns MD for each of the studied BRAF structures. The equilibrium ensembles and average properties of the BRAF dimer complexes were obtained by aggregating simulations from all independent MD trajectories. All crystal structures were obtained from the Protein Data Bank (RCSB PDB www.rcsb.org) [**131**]. Structure preparation process included several important steps that were previously reported in detail in our studies of protein kinases [**77,109,124,132**] and molecular chaperones [**133,134**]. In this protocol, hydrogen atoms and missing residues were assigned using the WHATIF program [**135**]. The missing segments of the A-loop in the BRAF crystal structures were modeled and reconstructed with the ArchPRED program [**136**]. The initial protonation states were assigned using the WHATIF program **and** optimized with the aid of the *H*++ web server [**137**]. The ligand charges and parameters were derived using a standard protocol that was described in detail in our recent studies [77, **134**]. According to this procedure, the crystallographic inhibitor conformations were initially optimized at the B3LYP/6-31G level using the Gaussian 09 package [**138**]. The charge parameters for the BRAF inhibitors were assigned initially by the ParamChem web server [**139]** and subsequently refined through the restrained electrostatic potential charge fitting procedure (RESP) [**140**]. The force field parameters for the BRAF inhibitors were obtained using a VMD plugin ffTK [**141**]. MD simulations were carried out using NAMD 2.6 package [**142**] with the CHARMM22 force field [**143,144**] and the explicit TIP3P water model [**145**]. The employed MD protocol was previously reported in detail in our studies of protein kinases [**77,109,124**] and molecular chaperones [**133,134**]. The initial structures were solvated in a water box with the buffering distance of 10 Å. Long-range nonbonded van der Waals interactions were computed using an atom-based cutoff of 12Å with switching van der Waals potential beginning at 10Å. The smooth particle mesh Ewald (PME) method [**146**] was employed to treat the long-range electrostatics. The simulation steps preceding the production period are consistent with our recent studies and were detailed previously [**77,134**]. An NPT production simulation was run on the equilibrated structures for 500 ns (or 200 ns for shorter simulations) keeping the temperature at 300 K and constant pressure (1 atm) using Langevin piston coupling algorithm.

The elastic network-based GNM approach [**103,104**] was used to describe collective motions and functional dynamics of the BRAF dimers. The formulation and implementation of this approach was previusly detailed in our recent studies [**77,134**]. In a nutshell, the protein structure is described as an isotropic fluctuating network of *N* residue nodes, each represented by the respective alpha-carbon atom. The protein topology is described by N × *N* Kirchhoff matrix of inter-residue contacts, **Γ**. The diagonal elements represent the coordination number of each residue. The pairs of residues located within an interaction cutoff distance *r*_*c*_ = 7.0 Å are connected by springs with a uniform spring constant γ. The GNM modes are determined by diagonalization of the Kirchhoff matrix, Γ = **UΛU**^**T**^.

$${\langle \Delta R}_{i}\cdot \Delta R_{j}\rangle =\frac{3k_{B}T}{\gamma }{\left[\Gamma^{\text{-}1}\right]}_{ij}=\frac{3k_{B}T}{\gamma }{\left[U\Lambda U^{T}\right]}_{ij}=\frac{3k_{B}T}{\gamma }\sum_{k=1}^{N-1}\lambda_{k}^{\text{-}1}{\left[u_{k}u_{k}^{T}\right]}_{ij}$$ (1)

Here, **U** is a unitary matrix, **U**^*T*^ = **U**^−1^ of the eigenvectors **u**_**k**_ of **Γ** and **Λ** is the diagonal matrix of eigenvalues **λ**_**k**_. The elements of the *k*th eigenvector **u**_**k**_ describe the displacements of the residues along the *k*th mode coordinate, and the *k*th eigenvalue, **λ**_**k**_, scales with the frequency of the *k*th mode, where 1 ≤ *k* ≤ *N*– 1. *K*_B_ is the Boltzmann constant, T is the absolute temperature. The mean square fluctuations of a residue are evaluated as a sum over the contributions of all modes. The fluctuation of the *i*^th^ atomic degree of freedom along the eigenvector **u**_**k**_ reflects the mobility of residue *i* in the *k*th mode.

$$\left\langle {\left(\Delta R_{i}\right)}^{2}\right\rangle =\frac{3k_{B}T}{\gamma }\sum_{k=1}^{N-1}{\left(\lambda_{k}^{\text{-}1}u_{k}u_{k}^{T}\right)}_{ii}$$ (2)

Conformational mobility profiles in the essential space of low frequency modes were obtained using the oGNM web server [**104**].

### Binding Free Energy Analysis

The binding free energy was calculated using the MM-GBSA approach [**115,116**]. In this model, the free energy of binding between an inhibitor and the BRAF kinase was evaluated as follows:

$$\Delta G_{bind}=<\Delta G_{MM}>+<\Delta G_{solv}>-<T\Delta S>$$ (3)

The gas-phase contribution <Δ*G*_*MM*_> to the binding free energy is the difference in the molecular mechanics energy of the complex and the isolated protein and ligand. This contribution is the sum of the differences in the intramolecular energies Δ*E*_intra_, the van der Waals interaction energy Δ*E*_*vdw*_, and the electrostatic interaction energy Δ*E*_*elec*_:

$$<\Delta G_{MM}>=\Delta E_{\mathrm{intra}}+\Delta E_{vdw}+\Delta E_{elec}$$ (4)

$$E_{\mathrm{intra}}=E_{bond}+E_{vdw}+E_{elec}$$ (5)

where *E*_*bond*_ is the energy of the bonded terms (bonds, angles, dihedral angles, and improper angles) of a given molecule; *E*_*vdw*_ is the van der Waals energy of the molecule; and *E*_*elec*_ is the electrostatic energy of the molecule. Δ*G*_*solv*_ is the solvation free energy, and −*T*Δ*S* is an entropy term.

The solvation free energy Δ*G*_*solv*_ is the difference between the solvation energy of the complex and solvation free energies of the isolated protein and ligand. This term is computed as the sum of nonpolar and polar terms:

$$\Delta G_{solv}=\Delta G_{solv}^{np}+\Delta G_{solv}^{elec}$$ (6)

The nonpolar contribution is evaluated from the solvent accessible surface area (SASA) model as $\Delta G_{solv}^{np}=\sigma \;*\;SASA$ +β where *σ* = 0.00542 kcal/ (mol*Ǻ^2^) and β = 0.92 kcal/mol. The electrostatic contribution to the solvation free energy of the system was estimated using the analytical generalized Born (GB) model. The entropy term includes contributions of translational Δ*S*_*trans*_, rotational Δ*S*_*rot*_ and vibrational Δ*S*_*vib*_ components:

$$\Delta S=\Delta S_{trans}+\Delta S_{rot}+\Delta S_{vib}$$ (7)

The vibrational entropy terms were computed using normal mode analysis in the VIBRAN module of the CHARMM program. All energy terms are calculated using MD trajectories of the complexes, which is followed by separation of the complexes into isolated protein and ligand structures and subsequent minimization of the isolated molecules. Computational alanine scanning [**117,118**] was performed by mutating the binding site residues and computing the binding free energy for the mutated system using 10,000 snapshots from 500 ns MD trajectory of the original complex. A single trajectory variation of the MM-GBSA approach was employed in which MD simulations of the BRAF complexes were followed by separation of the individual snapshots into isolated protein and ligand conformations. This protocol has been proven to be robust and efficient in binding free energy computations of the ligand-protein complexes, avoiding uncertainties and reducing cancellation errors associated with the differences in the intramolecular energies [**147**].

### Mutual Information Model and Coevolutionary Analysis

Coevolutionary dependencies of BRAF residues were evaluated using MI model and MISTIC approach [**95**]. The details of the MI model employed in this study were reported in our recent study of protein kinases [**124**]. The following MI parameters were computed: the Kullback-Leibler conservation score, a cumulative mutual information score (cMI), and a proximity mutual information score (pMI). The Kullback-Leibler (KL) score *KLConsScore* measures sequence conservation in the protein kinase family. For each column of the MSA, the KL conservation is calculated according to the following formula:

$$KLConsScore_{i}=\sum_{i=1}^{N}\ln\frac{P(i)}{Q(i)}$$ (8)

Here, *P*(*i*) is the frequency of amino acid *i* in that position and *Q*(*i*) is the background frequency of the amino acid in nature calculated using an amino acids background frequency distribution obtained from the UniProt database. cMI is a sequence-based MI score per residue position that estimates the contribution of a given residue to the MI network. cMI is calculated as the sum of MI values above a threshold t = 6.5 for every pair in which a particular residue of interest appears [**95**].

$$cMI_{x}=\sum_{y,MI(x,y)>t}MI_{\left(x,y\right)}$$ (9)

pMI score is a structure-based MI metric that is defined as the average of cMI scores of all the residues within a given distance from a given residue in the protein structure. The distance between each pair of residues was calculated as the shortest distance between any two heavy atoms that belong to each of these two positions. The threshold t = 5 Å is used to define structural proximity of a residue:

$$pMI_{x}=\frac{1}{N}\sum_{d(x,y),t}cMI_{\left(x,y\right)}$$ (10)

pMI scores of protein residues in the BRAF dime complexes were computed using ensemble-based definition of local residue proximity. In this dynamics-based model, the amount of mutual information shared by a given residue with the spatially close residues was averaged over 10,000 conformations from the MD trajectories of the BRAF dimers.

### Modeling of the Residue Interaction Networks: Communication Residue Propensities and Allosteric Pathways

In the construction of a protein structure network, a graph-based model of protein topological connectivity is used where residues are considered to be network nodes and are connected by edges representing residue interactions. The details of the graph construction using a particular interaction cut-off strength (*I*_min_) were extensively discussed in the initial reports [**84, 85**] and our previous studies [**77,109,124**]. We expanded the original model as the network edges that define residue connectivity are weighted based on both cross-correlation dynamic fluctuation and coevolutionary dependencies measured by the MI scores. The weight *w*_*ij*_ of a particular edge is defined as the element of a matrix **r**_*MI*_(**x**_*i*_,**x**_*j*_) measuring the dynamic and coevolutionary correlations for each pair of residues [**148,149**]. The length of the edge that connects nodes *i* and *j* is calculated from the corresponding generalized correlation coefficient as *w*_*ij*_ = −log[**r**_*MI*_(**x**_*i*_,**x**_*j*_)]. The weighted graph model defines a residue interaction network that favors a global flow of information through edges between residues associated with dynamics correlations and coevolutionary dependencies, which may be important for allosteric communication in the protein structure. The ensemble of shortest paths is determined from matrix of communication distances by the Floyd-Warshall algorithm [**150**] that compares all possible paths between each pair of residue nodes.

Using the constructed protein structure network, we compute the residue-based centrality parameter. The centrality of residue *i* is determined as its network betweenness computed as a fraction of the shortest paths between all pairs of residues that pass through residue *i*:

$$C_{b}(n_{i})=\sum_{j<k}^{N}\frac{g_{jk}(i)}{g_{jk}}$$ (11)

where *g*_*jk*_ denotes the number of shortest paths connecting *j* and *k*, and *g*_*jk*_(*i*) is the number of shortest paths between residues *j* and *k* that navigate through the node *n*_*i*_. Residues that populate a significant portion of the shortest paths connecting all residue pairs are characterized by high betweenness values (high residue centrality). For each node *n*, the betweenness value can be normalized by the number of node pairs that exclude node *n* which is given as (*N* - 1)(*N* - 2) / 2, where *N* is the total number of nodes in the connected component that node *n* belongs to. The normalized betweenness of residue *i* can be expressed as follows:

$$C_{b}(n_{i})=\frac{1}{\left(N-1)(N-2\right)}\sum_{\begin{array}{l} j<k\\ j\neq i\neq k \end{array}}^{N}\frac{g_{jk}(i)}{g_{jk}}$$ (12)

*g*_*jk*_ is the number of shortest paths between residues *j* and k; *g*_*jk*_(*i*) is the fraction of these shortest paths that pass through residue *i*.

We also computed communication propensities (CP) of protein residues defined as average variations of the effective “distance” metric that measures both distance fluctuations and variations in the pMI scores between a given residue and all other residues. Small fluctuations and respectively large CP values for a given residue are associated with high communication propensities and signal a high level of dynamic and coevolutionary cooperativity. For each residue, CP metric is evaluated as follows:

$$CP_{i}{}^{}=\frac{3k_{B}T}{\begin{array}{l} \left\langle w_{1}{\left(d_{i}-\left\langle d_{i}\right\rangle \right)}^{2}+w_{2}{\left(\Delta pM_{i}-\left\langle \Delta pM_{i}\right\rangle \right)}^{2}\right\rangle \end{array}}$$ (13)

$$d_{i}={\left\langle d_{ij}\right\rangle }_{j*}$$ (14)

$$\Delta pM_{i}={\left\langle \Delta pM_{ij}\right\rangle }_{j*}$$ (15)

where *d*_*ij*_ is the instantaneous distance between residue *i* and residue *j*, Δ*pM*_*ij*_ is the respective difference between pMI scores of residues *i* and *j* at the current 3D positions *x*_*i*_ and *x*_*i*_; *k*_*B*_ is the Boltzmann constant, *T* = 300K. 〈 〉 denotes an average taken over the MD simulation trajectory and *d*_*i*_ = 〈*d*_*ij*_〉_j*_ is the average distance from residue *i* to all other residues in the protein (the sum over j_*_ implies the exclusion of the atoms that belong to the residue *i*). Δ*pM*_*i*_ is the average difference in *pM*_*i*_ scores.

In the expression (13), *w*_1_ and *w*_2_ are weighting factors that are adjusted to optimize efficiency of the residue interaction network. The network efficiency is related to the average length of shortest paths among residue nodes. The length of a path *L*(*n*_*i*_,*n*_*j*_) between distant nodes *n*_*i*_ and *n*_*j*_ is the sum of the edge weights between the consecutive nodes (*n*_*k*_,*n*_*l*_) along the path:

$$L(n_{i},n_{j})=\sum_{kl}w(n_{k},n_{l})$$ (16)

## Supporting Information

S1 Fig Collective Dynamics of the BRAF-WT Monomer Structure. (A). The GMM-based conformational mobility profiles of the BRAF-WT monomer structure (pdb id 4WO5). The essential mobility of kinase residues is shown in the space of first two slowest modes (in green), the first three slowest modes (in red) and the first ten slowest modes (in blue). The position of the dimer interface residues in these profiles are highlighted in filled maroon-colored circles. (B) The inter-residue distance fluctuations map in the BRAF-WT is based on residue movements along the first three slow modes and derived using the ANM web server [**105**]. The large inter-residue fluctuations are shown in blue and small fluctuations are shown in red. (C) Structural maps of collective dynamics are based on fluctuations driven by the slowest three modes. The color gradient from red to blue indicates the increasing structural rigidity and refers to an average value over the backbone atoms in each residue.
(TIF)

S2 Fig The Slow Modes Shapes of the BRAF Dimer Structures. The GMM-based conformational mobility profiles along the three slowest modes are shown for the BRAF dimer complex with PLX4720 (pdb id 3C4C) (A), Dabrafenib (pdb id 4XV2) (B), Vemurafenib (pdb id 3OG7) (C), and PLX7904 (pdb id 4XV1) (D). The slow modes shapes are colored in green (first monomer) and red (second monomer).
(TIF)

S3 Fig Collectivity of the Slow Modes in the BRAF Dimer Structures. (A) The degree of collectivity of first 20 slow modes in the BRAF dimer structures is shown for the PLX4720-BRAF complex (in blue), Dabrafenib-BRAF complex (in red), Vemurafenib-BRAF complex (in green) and PLX7904-BRAF complex (in orange). High collectivity of the first five modes indicates that conformational movements in the BRAF structures can be described using a cumulative contribution of these slow modes. (B) Cumulative overlap of the GNM slow modes with the principal components describing conformational changes in the ensemble of BRAF crystal structures. This ensemble included all known BRAF dimer complexes with different classes of inhibitors as described in Fig 1. The softest five modes described ~90% of structural variations observed in the BRAF crystal structures.
(TIF)

S4 Fig Structural Mapping of the Dimer Interface Regions and R-spine Residues. (A) Structural mapping of the central interface section. The first monomer is shown in green ribbons and the second monomer is shown in cyan ribbons. The residues from the central section of the dimer interface (R506, R509, and F516) are annotated and shown as green spheres (first monomer) and cyan spheres (second monomer). The R-spine residues (V504, L505, F516, I572, H574, and F595) are annotated and shown as red spheres (first monomer) and blue spheres (second monomer). (B) Structural mapping of the secondary interface regions. The interface residues in this region (K475, W476, H577, R558, R562, D565, and Y566) are annotated and shown as green spheres (first monomer) and cyan spheres (second monomer).
(TIF)

## References

[1] Huse M, Kuriyan J. The conformational plasticity of protein kinases. Cell. 2002;109: 275–282. 12015977 10.1016/s0092-8674(02)00741-9

[2] Nolen B, Taylor S, Ghosh G. Regulation of protein kinases; controlling activity through activation segment conformation. Mol Cell. 2004;;15: 661–675. 15350212 10.1016/j.molcel.2004.08.024

[3] Taylor SS, Kornev AP. Protein kinases: evolution of dynamic regulatory proteins. Trends Biochem Sci. 2011;;36: 65–77. 20971646 10.1016/j.tibs.2010.09.006 PMC3084033

[4] Endicott JA, Noble ME, Johnson LN. The structural basis for control of eukaryotic protein kinases. Annu Rev Biochem. 2012;;81: 587–613. 22482904 10.1146/annurev-biochem-052410-090317

[5] Taylor SS, Keshwani MM, Steichen JM, Kornev AP. Evolution of the eukaryotic protein kinases as dynamic molecular switches. Philos Trans R Soc Lond B Biol Sci. 2012;;367: 2517–2528. 22889904 10.1098/rstb.2012.0054 PMC3415842

[6] Taylor SS, Ilouz R, Zhang P, Kornev AP. Assembly of allosteric macromolecular switches: lessons from PKA. Nat Rev Mol Cell Biol. 2012;;13: 646–658. 22992589 10.1038/nrm3432 PMC3985763

[7] Artim SC, Mendrola JM, Lemmon MA. Assessing the range of kinase autoinhibition mechanisms in the insulin receptor family. Biochem J. 2012;;448: 213–220. 22992069 10.1042/BJ20121365 PMC3492919

[8] Oruganty K, Kannan N. Design principles underpinning the regulatory diversity of protein kinases. Philos Trans R Soc Lond B Biol Sci. 2012;;367: 2529–2539. 22889905 10.1098/rstb.2012.0015 PMC3415841

[9] Taylor SS, Zhang P, Steichen JM, Keshwani MM, Kornev AP. PKA: lessons learned after twenty years. Biochim Biophys Acta, 2013;1834: 1271–1278. 23535202 10.1016/j.bbapap.2013.03.007 PMC3763834

[10] Meharena HS, Chang P, Keshwani MM, Oruganty K, Nene AK, Kannan N, et al Deciphering the structural basis of eukaryotic protein kinase regulation. PLoS Biol. 2013;;11: e1001680 10.1371/journal.pbio.1001680 24143133 PMC3797032

[11] Hantschel O. Structure, regulation, signaling, and targeting of Abl kinases in cancer. Genes Cancer. 2012;;3: 436–446. 23226581 10.1177/1947601912458584 PMC3513796

[12] Panjarian S, Iacob RE, Chen S, Engen JR, Smithgall TE. Structure and dynamic regulation of Abl kinases. J Biol Chem. 2013;;288: 5443–5450. 23316053 10.1074/jbc.R112.438382 PMC3581414

[13] Lemmon MA, Schlessinger J. Cell signaling by receptor tyrosine kinases. Cell. 2010;;141: 1117–11134. 20602996 10.1016/j.cell.2010.06.011 PMC2914105

[14] Roskoski R Jr. The ErbB/HER family of protein-tyrosine kinases and cancer. Pharmacol Res. 2014;;79: 34–74. 24269963 10.1016/j.phrs.2013.11.002

[15] Bayliss R, Fry A, Haq T, Yeoh S. On the molecular mechanisms of mitotic kinase activation. Open Biol. 2012;;2: 120136 10.1098/rsob.120136 23226601 PMC3513839

[16] Dodson CA, Haq T, Yeoh S, Fry AM, Bayliss R. The structural mechanisms that underpin mitotic kinase activation. Biochem Soc Trans. 2013;;41: 1037–1041. 23863175 10.1042/BST20130066

[17] Cowan-Jacob SW, Jahnke W, Knapp S. Novel approaches for targeting kinases: allosteric inhibition, allosteric activation and pseudokinases. Future Med Chem. 2014;; 6: 541–561. 24649957 10.4155/fmc.13.216

[18] Cowan-Jacob SW. Structural biology of protein tyrosine kinases. Cell Mol Life Sci. 2006;;63: 2608–2625. 10.1007/s00018-006-6202-8 17041812 PMC11136174

[19] Roskoski R Jr. A historical overview of protein kinases and their targeted small molecule inhibitors. Pharmacol Res. 2015;;100: 1–23. 26207888 10.1016/j.phrs.2015.07.010

[20] Levinson NM, Kuchment O, Shen K, Young MA, Koldobskiy M, Karplus M, et al A SRC-like inactive conformation in the ABL tyrosine kinase domain. PLoS Biol. 2006;;4: 0753–0767. 10.1371/journal.pbio.0040144 PMC1450098 16640460

[21] Jura N, Zhang X, Endres NF, Seeliger MA, Schindler T, Kuriyan J. Catalytic control in the EGF receptor and its connection to general kinase regulatory mechanisms. Mol Cell. 2011;;42: 9–22. 21474065 10.1016/j.molcel.2011.03.004 PMC3175429

[22] Kornev AP, Haste NM, Taylor SS, Eyck LF. Surface comparison of active and inactive protein kinases identifies a conserved activation mechanism. Proc Natl Acad Sci U S A. 2006;;103: 17783–17788. 17095602 10.1073/pnas.0607656103 PMC1693824

[23] Kornev AP, Taylor SS, Ten Eyck LF. A helix scaffold for the assembly of active protein kinases. Proc Natl Acad Sci U S A. 2008;;105: 14377–14382. 18787129 10.1073/pnas.0807988105 PMC2533684

[24] Hu J, Ahuja LG, Meharena HS, Kannan N, Kornev AP, Taylor SS, et al Kinase regulation by hydrophobic spine assembly in cancer. Mol Cell Biol. 2015;;35: 264–276. 25348715 10.1128/MCB.00943-14 PMC4295384

[25] Ferguson KM. Structure-based view of epidermal growth factor receptor regulation. Annu Rev Biophys. 2008;;37: 353–373. 10.1146/annurev.biophys.37.032807.125829 18573086 PMC2745238

[26] Lemmon MA. Ligand-induced ErbB receptor dimerization. Exp Cell Res. 2009;; 315: 638–648. 10.1016/j.yexcr.2008.10.024 PMC2667204 19038249

[27] Bose R, Zhang X. The ErbB kinase domain: structural perspectives into kinase activation and inhibition. Exp Cell Res. 2009;;315: 649–658. 18761339 10.1016/j.yexcr.2008.07.031 PMC2668223

[28] Roskoski R Jr. ErbB/HER protein-tyrosine kinases: Structures and small molecule inhibitors. Pharmacol Res. 2014;;87: 42–59. 24928736 10.1016/j.phrs.2014.06.001

[29] Endres NF, Barros T, Cantor AJ, Kuriyan J. Emerging concepts in the regulation of the EGF receptor and other receptor tyrosine kinases. Trends Biochem Sci. 2014;; 39: 437–446. 25242369 10.1016/j.tibs.2014.08.001

[30] Kovacs E, Zorn JA, Huang Y, Barros T, Kuriyan J. A structural perspective on the regulation of the epidermal growth factor receptor. Annu Rev Biochem. 2015;;84: 739–764. 25621509 10.1146/annurev-biochem-060614-034402 PMC4452390

[31] Rajakulendran T, Sahmi M, Lefrancois M, Sicheri F, Therrien M. A dimerization-dependent mechanism drives RAF catalytic activation. Nature. 2009;;461: 542–545. 19727074 10.1038/nature08314

[32] Roskoski R Jr. RAF protein-serine/threonine kinases: structure and regulation. Biochem Biophys Res Commun. 2010;; 399: 313–317. 10.1016/j.bbrc.2010.07.092 20674547

[33] Lavoie H, Li JJ, Thevakumaran N, Therrien M, Sicheri F. Dimerization-induced allostery in protein kinase regulation. Trends Biochem Sci. 2014;;39: 475–486. 25220378 10.1016/j.tibs.2014.08.004

[34] Jambrina PG, Bohuszewicz O, Buchete NV, Kolch W, Rosta E. Molecular mechanisms of asymmetric RAF dimer activation. Biochem Soc Trans. 2014;; 42: 784–790. 25109958 10.1042/BST20140025

[35] Kornev AP, Taylor SS. Dynamics-driven allostery in protein kinases. Trends Biochem Sci. 2015;;40: 628–647. 26481499 10.1016/j.tibs.2015.09.002 PMC4630092

[36] Shaw AS, Kornev AP, Hu J, Ahuja LG, Taylor SS. Kinases and pseudokinases: lessons from RAF. Mol Cell Biol. 2014;;34: 1538–1546. 24567368 10.1128/MCB.00057-14 PMC3993607

[37] Hu J, Stites EC, Yu H, Germino EA, Meharena HS, Stork PJ, et al Allosteric activation of functionally asymmetric RAF kinase dimers. Cell. 2013;;154: 1036–1046. 23993095 10.1016/j.cell.2013.07.046 PMC3844432

[38] Thevakumaran N, Lavoie H, Critton DA, Tebben A, Marinier A, et al Crystal structure of a BRAF kinase domain monomer explains basis for allosteric regulation. Nat Struct Mol Biol. 2015;;22: 37–43. 25437913 10.1038/nsmb.2924

[39] Lavoie H, Thevakumaran N, Gavory G, Li JJ, Padeganeh A, Guiral S, et al Inhibitors that stabilize a closed RAF kinase domain conformation induce dimerization. Nat Chem Biol. 2013;; 9: 428–436. 23685672 10.1038/nchembio.1257 PMC4954776

[40] Poulikakos PI, Zhang C, Bollag G, Shokat KM, Rosen N. RAF inhibitors transactivate RAF dimers and ERK signalling in cells with wild-type BRAF. Nature. 2010;;464: 427–430. 20179705 10.1038/nature08902 PMC3178447

[41] Hatzivassiliou G, Song K, Yen I, Brandhuber BJ, Anderson DJ, Alvarado R, et al RAF inhibitors prime wild-type RAF to activate the MAPK pathway and enhance growth. Nature. 2010;;464: 431–435. 20130576 10.1038/nature08833

[42] Heidorn SJ, Milagre C, Whittaker S, Nourry A, Niculescu-Duvas I, Dhomen N, et al Kinase-dead BRAF and oncogenic RAS cooperate to drive tumor progression through CRAF. Cell. 2010;;140: 209–221. 20141835 10.1016/j.cell.2009.12.040 PMC2872605

[43] Cichowski K, Jänne PA. Drug discovery: inhibitors that activate. Nature. 2010;; 464: 358–359. 10.1038/464358a 20237552

[44] Holderfield M, Nagel TE, Stuart DD. Mechanism and consequences of RAF kinase activation by small-molecule inhibitors. Br J Cancer. 2014;;111: 640–645. 24642617 10.1038/bjc.2014.139 PMC4134487

[45] Dar AC, Shokat KM. The evolution of protein kinase inhibitors from antagonists to agonists of cellular signaling. Annu Rev Biochem. 2011;;80: 769–795. 21548788 10.1146/annurev-biochem-090308-173656

[46] Fabbro D, Cowan-Jacob S, Möbitz H, Martiny-Baron G. Targeting cancer with small-molecular-weight kinase inhibitors. Methods Mol Biol. 2012;;795: 1–34. 21960212 10.1007/978-1-61779-337-0_1

[47] Fabbro D. 25 years of small molecular weight kinase inhibitors: potentials and limitations. Mol Pharmacol. 2015;;87: 766–775. 25549667 10.1124/mol.114.095489

[48] Wu P, Nielsen TE, Clausen MH. FDA-approved small-molecule kinase inhibitors. Trends Pharmacol Sci. 2015;;36: 422–439. 25975227 10.1016/j.tips.2015.04.005

[49] Wu P, Clausen MH, Nielsen TE. Allosteric small-molecule kinase inhibitors. Pharmacol Ther. 2015;;156: 59–68. 26478442 10.1016/j.pharmthera.2015.10.002

[50] Roskoski R Jr. Classification of small molecule protein kinase inhibitors based upon the structures of their drug-enzyme complexes. Pharmacol Res. 2016;;103: 26–48. 26529477 10.1016/j.phrs.2015.10.021

[51] King AJ, Patrick DR, Batorsky RS, Ho ML, Do HT, Zhang SY, et al Demonstration of a genetic therapeutic index for tumors expressing oncogenic BRAF by the kinase inhibitor SB-590885. Cancer Res. 2006;;66: 11100–11105. 10.1158/0008-5472.CAN-06-2554 17145850

[52] Haling JR, Sudhamsu J, Yen I, Sideris S, Sandoval W, Phung W, et al Structure of the BRAF-MEK complex reveals a kinase activity independent role for BRAF in MAPK signaling. Cancer Cell. 2014;;26: 402–413. 25155755 10.1016/j.ccr.2014.07.007

[53] Wan PT, Garnett MJ, Roe SM, Lee S, Niculescu-Duvaz D, Good VM, et al Mechanism of activation of the RAF-ERK signaling pathway by oncogenic mutations of B-RAF. Cell. 2004;;116: 855–867. 15035987 10.1016/s0092-8674(04)00215-6

[54] Peng SB, Henry JR, Kaufman MD, Lu WP, Smith BD, Vogeti S, et al Inhibition of RAF isoforms and active dimers by LY3009120 leads to anti-tumor activities in RAS or BRAF mutant cancers. Cancer Cell. 2015;;28: 384–398. 26343583 10.1016/j.ccell.2015.08.002

[55] Okaniwa M, Hirose M, Arita T, Yabuki M, Nakamura A, Takagi T, et al Discovery of a selective kinase inhibitor (TAK-632) targeting pan-RAF inhibition: design, synthesis, and biological evaluation of C-7-substituted 1,3-benzothiazole derivatives. J Med Chem. 2013;;56: 6478–6494. 23906342 10.1021/jm400778d

[56] Nakamura A, Arita T, Tsuchiya S, Donelan J, Chouitar J, Carideo E, et al Antitumor activity of the selective pan-RAF inhibitor TAK-632 in BRAF inhibitor-resistant melanoma. Cancer Res. 2013;;73: 7043–7055. 10.1158/0008-5472.CAN-13-1825 24121489

[57] Williams TE, Subramanian S, Verhagen J, McBride CM, Costales A, Sung L, et al Discovery of RAF265: A Potent mut-B-RAF Inhibitor for the Treatment of Metastatic Melanoma. ACS Med Chem Lett. 2015;;6: 961–965. 26396681 10.1021/ml500526p PMC4569875

[58] Wenglowsky S, Moreno D, Laird ER, Gloor SL, Ren L, Risom T, et al Pyrazolopyridine inhibitors of B-Raf(V600E). Part 4: rational design and kinase selectivity profile of cell potent type II inhibitors. Bioorg Med Chem Lett. 2012;;22: 6237–6241. 22954737 10.1016/j.bmcl.2012.08.007

[59] Tsai J, Lee JT, Wang W, Zhang J, Cho H, Mamo S, et al Discovery of a selective inhibitor of oncogenic B-Raf kinase with potent antimelanoma activity. Proc Natl Acad Sci U S A. 2008;;105: 3041–3046. 18287029 10.1073/pnas.0711741105 PMC2268581

[60] Bollag G, Hirth P, Tsai J, Zhang J, Ibrahim PN, Cho H, et al Clinical efficacy of a RAF inhibitor needs broad target blockade in BRAF-mutant melanoma. Nature. 2010;;467: 596–599. 20823850 10.1038/nature09454 PMC2948082

[61] Waizenegger IC, Baum A, Steurer S, Stadtmuller H, Bader G, Schaaf O, et al A novel RAF kinase inhibitor with DFG-out binding mode: High efficacy in BRAF-mutant tumor xenograft models in the absence of normal tissue hyperproliferation. Mol Cancer Ther. 2016;;15: 354–365. 10.1158/1535-7163.MCT-15-0617 26916115

[62] Zhang C, Spevak W, Zhang Y, Burton EA, Ma Y, Habets G, et al RAF inhibitors that evade paradoxical MAPK pathway activation. Nature. 2015;;526: 583–586. 26466569 10.1038/nature14982

[63] Le K, Blomain ES, Rodeck U, Aplin AE. Selective RAF inhibitor impairs ERK1/2 phosphorylation and growth in mutant NRAS, vemurafenib-resistant melanoma cells. Pigment Cell Melanoma Res. 2013;;26: 509–517. 23490205 10.1111/pcmr.12092 PMC3695051

[64] Arora R, Di Michele M, Stes E, Vandermarliere E, Martens L, Gevaert K, et al Structural investigation of B-Raf paradox breaker and inducer inhibitors. J Med Chem. 2015;;58: 1818–1831. 25611072 10.1021/jm501667n

[65] Dixit A, Verkhivker GM. Computational modeling of allosteric communication reveals organizing principles of mutation-induced signaling in ABL and EGFR kinases. PLoS Comput Biol. 2011;;7: e1002179 10.1371/journal.pcbi.1002179 21998569 PMC3188506

[66] Telesco SE, Shih AJ, Jia F, Radhakrishnan R. A multiscale modeling approach to investigate molecular mechanisms of pseudokinase activation and drug resistance in the HER3/ErbB3 receptor tyrosine kinase signaling network. Mol Biosyst. 2011;;7: 2066–280. 10.1039/c0mb00345j 21509365 PMC3138520

[67] Wan S, Coveney PV. Molecular dynamics simulation reveals structural and thermodynamic features of kinase activation by cancer mutations within the epidermal growth factor receptor. J Comput Chem. 2011;;32: 2843–2852. 10.1002/jcc.21866 21717480

[68] Shan Y, Eastwood MP, Zhang X, Kim ET, Arkhipov A, Dror RO, et al Oncogenic mutations counteract intrinsic disorder in the EGFR kinase and promote receptor dimerization. Cell. 2012;;149: 860–870. 22579287 10.1016/j.cell.2012.02.063

[69] Shan Y, Arkhipov A, Kim ET, Pan AC, Shaw DE. Transitions to catalytically inactive conformations in EGFR kinase. Proc Natl Acad Sci U S A. 2013;;110: 7270–7275. 23576739 10.1073/pnas.1220843110 PMC3645566

[70] Sutto L, Gervasio FL. Effects of oncogenic mutations on the conformational free-energy landscape of EGFR kinase. Proc Natl Acad Sci U S A. 2013;;110: 10616–10621. 23754386 10.1073/pnas.1221953110 PMC3696764

[71] Endres NF, Das R, Smith AW, Arkhipov A, Kovacs E, Huang Y, et al Conformational coupling across the plasma membrane in activation of the EGF receptor. Cell. 2013;;152: 543–556. 23374349 10.1016/j.cell.2012.12.032 PMC3718647

[72] Moretti S, De Falco V, Tamburrino A, Barbi F, Tavano M, Avenia N, et al Insights into the molecular function of the inactivating mutations of B-Raf involving the DFG motif. Biochim Biophys Acta. 2009;;1793: 1634–1645. 19735675 10.1016/j.bbamcr.2009.09.001

[73] Caballero J, Alzate-Morales JH, Vergara-Jaque A. Investigation of the differences in activity between hydroxycycloalkyl N1 substituted pyrazole derivatives as inhibitors of B-Raf kinase by using docking, molecular dynamics, QM/MM, and fragment-based de novo design: study of binding mode of diastereomer compounds. J Chem Inf Model. 2011;;51: 2920–2931. 10.1021/ci200306w 22011048

[74] Li Y, Han C, Wang J, Yang Y, Zhang J, Zhang S, Yang L. Insight into the structural features of pyrazolopyrimidine- and pyrazolopyridine-based B-Raf(V600E) kinase inhibitors by computational explorations. Chem Biol Drug Des. 2014;83:643–655. 24373283 10.1111/cbdd.12276

[75] Coronel L, Granadino-Roldán JM, Pinto M, Tomas MS, Pujol MD, Rubio-Martinez J. Insight into the binding of DFG-out allosteric inhibitors to B-Raf kinase using molecular dynamics and free energy calculations. Curr Comput Aided Drug Des. 2015;;11: 124–136. 26135342 10.2174/1573409911666150702100245

[76] Marino KA, Sutto L, Gervasio FL. The effect of a widespread cancer-causing mutation on the inactive to active dynamics of the B-Raf kinase. J Am Chem Soc. 2015;;137: 5280–5283. 25868080 10.1021/jacs.5b01421

[77] Verkhivker GM Molecular dynamics simulations and modelling of the residue interaction networks in the BRAF kinase complexes with small molecule inhibitors: probing the allosteric effects of ligand-induced kinase dimerization and paradoxical activation. Mol BioSyst. 2016;;12: 3146–3165. 27481329 10.1039/c6mb00298f

[78] Li C, Ma N, Wang Y, Wang Y, Chen G. Molecular dynamics simulation studies on the positive cooperativity of the Kemptide substrate with protein kinase A induced by the ATP ligand. J Phys Chem B. 2014;;118: 1273–1287. 24456306 10.1021/jp411111g

[79] Bren U, Oostenbrink CJ. Cytochrome P450 3A4 inhibition by ketoconazole: Tackling the problem of ligand cooperativity using molecular dynamics simulations and free-energy calculations. J Chem Inf Model. 2012;;52: 1573–1582. 10.1021/ci300118x 22587011

[80] Dam TK, Roy R, Pagé D, Brewer CF. Negative cooperativity associated with binding of multivalent carbohydrates to lectins. Thermodynamic analysis of the "multivalency effect". Biochemistry. 2002;;41: 1351–1358. 11802737 10.1021/bi015830j

[81] Stevens SY, Sanker S, Kent C, Zuiderweg ER. Delineation of the allosteric mechanism of a cytidylyltransferase exhibiting negative cooperativity. Nat Struct Biol. 2001;;8: 947–952. 11685240 10.1038/nsb1101-947

[82] Nesmelova IV, Ermakova E, Daragan VA, Pang M, Menéndez M, Lagartera L, et al Lactose binding to galectin-1 modulates structural dynamics, increases conformational entropy, and occurs with apparent negative cooperativity. J Mol Biol. 2010;;397: 1209–1230. 20184898 10.1016/j.jmb.2010.02.033

[83] Atilgan AR, Akan P, Baysal C. Small-world communication of residues and significance for protein dynamics. Biophys J. 2004;;86: 85–91. 14695252 10.1016/S0006-3495(04)74086-2 PMC1303839

[84] Brinda KV, Vishveshwara S. A network representation of protein structures: implications for protein stability. Biophys J. 2005;;89: 4159–4170. 16150969 10.1529/biophysj.105.064485 PMC1366981

[85] Vijayabaskar MS, Vishveshwara S Interaction energy based protein structure networks. Biophys J. 2010;;99: 3704–3715. 21112295 10.1016/j.bpj.2010.08.079 PMC2998601

[86] Amitai G, Shemesh A, Sitbon E, Shklar M, Netanely D, Venger I, et al Network analysis of protein structures identifies functional residues. J Mol Biol. 2004;; 344: 1135–1146. 10.1016/j.jmb.2004.10.055 15544817

[87] del Sol A, Fujihashi H, Amoros D, Nussinov R. Residues crucial for maintaining short paths in network communication mediate signaling in proteins. Mol Syst Biol. 2006;2: 2006.0019. 10.1038/msb4100063 PMC1681495 16738564

[88] Suel GM, Lockless SW, Wall MA, Ranganathan R. Evolutionarily conserved networks of residues mediate allosteric communication in proteins. Nat Struct Biol. 2003;10: 59–69. 12483203 10.1038/nsb881

[89] Halabi N, Rivoire O, Leibler S, Ranganathan R. Protein sectors: evolutionary units of three-dimensional structure. Cell. 2009;138: 774–786. 19703402 10.1016/j.cell.2009.07.038 PMC3210731

[90] McLaughlin RN, Poelwijk FJ, Raman A, Gosal WS, Ranganathan R. The spatial architecture of protein function and adaptation. Nature. 2012;491: 138–142. 23041932 10.1038/nature11500 PMC3991786

[91] Gloor GB, Martin LC, Wahl LM, Dunn SD. Mutual information in protein multiple sequence alignments reveals two classes of coevolving positions. Biochemistry. 2005;44: 7156–7165. 15882054 10.1021/bi050293e

[92] Aguilar D, Oliva B, Marino Buslje C. Mapping the mutual information network of enzymatic families in the protein structure to unveil functional features. PLoS One. 2012;7: e41430 10.1371/journal.pone.0041430 22848494 PMC3405127

[93] Buslje C, Teppa E, Di Doménico T, Delfino JM, Nielsen M. Networks of high mutual information define the structural proximity of catalytic sites: implications for catalytic residue identification. PLoS Comput Biol. 2010;6: e1000978 10.1371/journal.pcbi.1000978 21079665 PMC2973806

[94] Teppa E, Wilkins AD, Nielsen M, Buslje C. Disentangling evolutionary signals: conservation, specificity determining positions and coevolution. Implication for catalytic residue prediction. BMC Bioinformatics. 2012;13: 235. 22978315 10.1186/1471-2105-13-235 PMC3515339

[95] Simonetti FL, Teppa E, Chernomoretz A, Nielsen M, Marino Buslje C. MISTIC: Mutual information server to infer coevolution. Nucleic Acids Res. 2013;41: W8–W14. 23716641 10.1093/nar/gkt427 PMC3692073

[96] Chakrabarti S, Panchenko AR. Coevolution in defining the functional specificity. Proteins. 2009;75: 231–240. 18831050 10.1002/prot.22239 PMC2649964

[97] Chakrabarti S, Panchenko AR. Structural and functional roles of coevolved sites in proteins. PLoS One. 2010;5: e8591 10.1371/journal.pone.0008591 20066038 PMC2797611

[98] Zhao Y, Wang Y, Gao Y, Li G, Huang J. Integrated analysis of residue coevolution and protein structures capture key protein sectors in HIV-1 proteins. PLoS One. 2015;10: e0117506 10.1371/journal.pone.0117506 25671429 PMC4324911

[99] Xu F, Du P, Shen H, Hu H, Wu Q, Xie J, et al Correlated mutation analysis on the catalytic domains of serine/threonine protein kinases. PLoS One. 2009;4: e5913 10.1371/journal.pone.0005913 19526051 PMC2690836

[100] Jeon J, Nam HJ, Choi YS, Yang JS, Hwang J, Kim S. Molecular evolution of protein conformational changes revealed by a network of evolutionarily coupled residues. Mol Biol Evol. 2011;28: 2675–2685. 21470969 10.1093/molbev/msr094

[101] Freed DM, Park JH, Radhakrishnan R, Lemmon MA. Deletion mutations keep kinase inhibitors in the loop. Cancer Cell. 2016;29: 423–425. 27070691 10.1016/j.ccell.2016.03.017 PMC5028821

[102] Foster SA, Whalen DM, Özen A, Wongchenko MJ, Yin J, Yen I, et al Activation mechanism of oncogenic deletion mutations in BRAF, EGFR, and HER2. Cancer Cell. 2016;29: 477–493. 26996308 10.1016/j.ccell.2016.02.010

[103] Bahar I, Lezon TR, Yang LW, Eyal E. Global dynamics of proteins: bridging between structure and function. Annu Rev Biophys. 2010;39: 23–42. 20192781 10.1146/annurev.biophys.093008.131258 PMC2938190

[104] Yang LW, Rader AJ, Liu X, Jursa CJ, Chen SC, et al oGNM: online computation of structural dynamics using the Gaussian Network Model. Nucleic Acids Res. 2006;34: W24–W31. 16845002 10.1093/nar/gkl084 PMC1538811

[105] Eyal E, Lum G, Bahar I. The anisotropic network model web server at 2015 (ANM 2.0). Bioinformatics. 2015;31: 1487–1489. 25568280 10.1093/bioinformatics/btu847 PMC4410662

[106] Masterson LR, Shi L, Metcalfe E, Gao J, Taylor SS, Veglia G. Dynamically committed, uncommitted, and quenched states encoded in protein kinase A revealed by NMR spectroscopy. Proc Natl Acad Sci U S A. 2011;108: 6969–6974. 21471451 10.1073/pnas.1102701108 PMC3084134

[107] Masterson LR, Cembran A, Shi L, Veglia G. Allostery and binding cooperativity of the catalytic subunit of protein kinase A by NMR spectroscopy and molecular dynamics simulations. Adv Protein Chem Struct Biol. 2012;87: 363–89. 10.1016/B978-0-12-398312-1.00012-3 22607761 PMC3546502

[108] Keskin O. Comparison of full-atomic and coarse-grained models to examine the molecular fluctuations of c-AMP dependent protein kinase. J Biomol Struct Dyn. 2002;20: 333–345. 12437372 10.1080/07391102.2002.10506852

[109] James KA, Verkhivker GM. Structure-based network analysis of activation mechanisms in the ErbB family of receptor tyrosine kinases: the regulatory spine residues are global mediators of structural stability and allosteric interactions. PLoS One. 2014;9: e113488 10.1371/journal.pone.0113488 25427151 PMC4245119

[110] Yang L, Song G, Carriquiry A, Jernigan RL. Close correspondence between the motions from principal component analysis of multiple HIV-1 protease structures and elastic network modes. Structure. 2008;16: 321–330. 18275822 10.1016/j.str.2007.12.011 PMC2350220

[111] Meireles L, Gur M, Bakan A, Bahar I. Pre-existing soft modes of motion uniquely defined by native contact topology facilitate ligand binding to proteins. Protein Sci. 2011;20: 1645–1658. 21826755 10.1002/pro.711 PMC3218357

[112] Yang LW, Bahar I. Coupling between catalytic site and collective dynamics: a requirement for mechanochemical activity of enzymes. Structure. 2005;13: 893–904. 15939021 10.1016/j.str.2005.03.015 PMC1489920

[113] Tobi D, Bahar I. Structural changes involved in protein binding correlate with intrinsic motions of proteins in the unbound state. Proc Natl Acad Sci U S A. 2005;102: 18908–18913. 16354836 10.1073/pnas.0507603102 PMC1323175

[114] Keskin O. Binding induced conformational changes of proteins correlate with their intrinsic fluctuations: a case study of antibodies. BMC Struct Biol. 2007;7: 31. 17509130 10.1186/1472-6807-7-31 PMC1888692

[115] Srinivasan J, Cheatham TE, Cieplak P, Kollman PA, Case DA. Continuum solvent studies of the stability of DNA, RNA and phosphoramidate-DNA helices. J Am Chem Soc 1998;120: 9401–9409.

[116] Kollman PA, Massova I, Reyes C, Kuhn B, Huo S, Chong L, et al Calculating structures and free energies of complex molecules: combining molecular mechanics and continuum models. Acc Chem Res. 2000;33: 889–897. 11123888 10.1021/ar000033j

[117] Massova I, Kollman PA. Computational alanine scanning to probe protein−protein interactions: a novel approach to evaluate binding free energies. J Am Chem Soc. 1999; 121: 8133–8143.

[118] Huo S, Massova I, Kollman PA. Computational alanine scanning of the 1:1 human growth hormone-receptor complex. J Comput Chem. 2002;23: 15–27. 11913381 10.1002/jcc.1153

[119] Choi J, Landrette SF, Wang T, Evans P, Bacchiocchi A, Bjornson R, et al Identification of PLX4032-resistance mechanisms and implications for novel RAF inhibitors. Pigment Cell Melanoma Res. 2014;27: 253–262. 24283590 10.1111/pcmr.12197 PMC4065135

[120] Basile KJ, Le K, Hartsough EJ, Aplin AE. Inhibition of mutant BRAF splice variant signaling by next-generation, selective RAF inhibitors. Pigment Cell Melanoma Res. 2014;27: 479–484. 109 24422853 10.1111/pcmr.12218 PMC3988223

[121] Popovych N, Sun S, Ebright RH, Kalodimos CG. Dynamically driven protein allostery. Nat Struct Mol Biol. 2006;13: 831–838. 16906160 10.1038/nsmb1132 PMC2757644

[122] Tzeng SR, Kalodimos CG. Protein activity regulation by conformational entropy. Nature. 2012; 488: 236–240. 22801505 10.1038/nature11271

[123] Yao Z, Torres NM, Tao A, Gao Y, Luo L, Li Q, et al BRAF mutants evade ERK-dependent feedback by different mechanisms that determine their sensitivity to pharmacologic inhibition. Cancer Cell. 2015;28: 370–383. 26343582 10.1016/j.ccell.2015.08.001 PMC4894664

[124] Tse A, Verkhivker GM. Molecular determinants underlying binding specificities of the ABL kinase Inhibitors: Combining alanine scanning of binding hot spots with network analysis of residue Interactions and coevolution. PLoS One. 2015;10: e0130203 10.1371/journal.pone.0130203 26075886 PMC4468085

[125] Sacquin-Mora S, Laforet E, Lavery R. Locating the active sites of enzymes using mechanical properties. Proteins. 2007;67: 350–359. 17311346 10.1002/prot.21353

[126] Sacquin-Mora S, Delalande O, Baaden M. Functional modes and residue flexibility control the anisotropic response of guanylate kinase to mechanical stress. Biophys J. 2010; 99: 3412–3419. 21081090 10.1016/j.bpj.2010.09.026 PMC2980708

[127] Blacklock K, Verkhivker G. Differential modulation of functional dynamics and allosteric interactions in the Hsp90-cochaperone complexes with p23 and Aha1: a computational study. PLoS One. 2013;8: e71936 10.1371/journal.pone.0071936 23977182 PMC3747073

[128] Kitano H. A robustness-based approach to systems-oriented drug design. Nat Rev Drug Discov. 2007;6: 202–210. 10.1038/nrd2195 17318209

[129] Pei J, Yin N, Ma X, Lai L. Systems biology brings new dimensions for structure-based drug design. J Am Chem Soc. 2014;136: 11556–11565. 25061983 10.1021/ja504810z

[130] Csermely P, Korcsmáros T, Kiss HJ, London G, Nussinov R. Structure and dynamics of molecular networks: a novel paradigm of drug discovery: a comprehensive review. Pharmacol Ther. 2013;138: 333–408. 23384594 10.1016/j.pharmthera.2013.01.016 PMC3647006

[131] Berman HM, Westbrook J, Feng Z, Gilliland G, Bhat TN, Weissig H, et al The Protein Data Bank. Nucleic Acids Res. 2000; 28: 235–242. 10592235 10.1093/nar/28.1.235 PMC102472

[132] Tse A, Verkhivker GM. Molecular dynamics simulations and structural network analysis of c-Abl and c-Src kinase core proteins: Capturing allosteric mechanisms and communication pathways from residue centrality. J Chem Inf Model. 2015;55: 1645–1662. 10.1021/acs.jcim.5b00240 26236953

[133] Stetz G, Verkhivker GM. Dancing through life: Molecular dynamics simulations and network-centric modeling of allosteric mechanisms in Hsp70 and Hsp110 chaperone proteins. PLoS One. 2015;10: e0143752 10.1371/journal.pone.0143752 26619280 PMC4664246

[134] Stetz G, Verkhivker GM. Probing allosteric inhibition mechanisms of the Hsp70 chaperone proteins using molecular dynamics simulations and analysis of the residue interaction networks. J Chem Inf Model. 2016;56:1490–1517. 27447295 10.1021/acs.jcim.5b00755

[135] Hekkelman ML, Te Beek TA, Pettifer SR, Thorne D, Attwood TK, Vriend G. WIWS: A protein structure bioinformatics web service collection. Nucleic Acids Res. 2010;38: W719–W723. 10.1093/nar/gkq453 20501602 PMC2896166

[136] Fernandez-Fuentes N, Zhai J, Fiser A. ArchPRED: a template based loop structure prediction server. Nucleic Acids Res. 2006;34: W173–W176. 16844985 10.1093/nar/gkl113 PMC1538831

[137] Anandakrishnan R, Aguilar B, Onufriev AV. H++ 3.0: Automating pK prediction and the preparation of biomolecular structures for atomistic molecular modeling and simulations. Nucleic Acids Res. 2012;40: W537–W541. 10.1093/nar/gks375 22570416 PMC3394296

[138] Frisch MJ, Trucks GW, Schlegel HB, Scuseria GE, Robb MA, Cheeseman JR, et al Gaussian09, Revision D.01; Gaussian Inc.: Wallingford CT, 2009.

[139] Vanommeslaeghe K, Raman EP, MacKerell AD Jr. Automation of the CHARMM General Force Field (CGenFF) II: assignment of bonded parameters and partial atomic charges. J Chem Inf Model. 2012;52: 3155–3168. 10.1021/ci3003649 23145473 PMC3528813

[140] Cieplak P, Cornell WD, Bayly CI, Kollman PA. Application of the multimolecule and multiconformational RESP methodology to biopolymers: charge derivation for DNA, RNA and proteins. J Comput Chem. 1995;16: 1357–1377.

[141] Mayne CG, Saam J, Schulten K, Tajkhorshid E, Gumbart JC. Rapid parameterization of small molecules using the force field toolkit. J Comput Chem. 2013;34: 2757–2770. 10.1002/jcc.23422 24000174 PMC3874408

[142] Phillips JC, Braun R, Wang W, Gumbart J, Tajkhorshid E, Villa E, et al Scalable molecular dynamics with NAMD. J Comput Chem. 2005; 26: 1781–1802. 16222654 10.1002/jcc.20289 PMC2486339

[143] MacKerell AD Jr, Bashford D, Bellott M, Dunbrack RL Jr, Evanseck JD, Field MJ, et al All-atom empirical potential for molecular modeling and dynamics studies of proteins. J Phys Chem B. 1998;102:3586–3616. 24889800 10.1021/jp973084f

[144] MacKerell AD Jr, Banavali N, Foloppe N. Development and current status of the CHARMM force field for nucleic acids. Biopolymers. 2001;56: 257–265. 10.1002/1097-0282(2000)56:4<257::AID-BIP10029>3.0.CO;2-W 11754339

[145] Jorgensen WL, Chandrasekhar J, Madura JD, Impey RW, Klein ML. Comparison of simple potential functions for simulating liquid water. J Chem Phys. 1983;79: 926–935.

[146] Essmann U, Perera L, Berkowitz ML, Darden T, Lee H, Pedersen LG. A smooth particle mesh Ewald method. J Chem Phys. 1995;103: 8577–8593.

[147] Hou T.; Wang J.; Li Y.; Wang W. Assessing the performance of the MM/PBSA and MM/GBSA methods. 1. The accuracy of binding free energy calculations based on molecular dynamics simulations. J Chem Inf Model. 2011;51: 69–82. 21117705 10.1021/ci100275a PMC3029230

[148] Kraskov A, Stogbauer H, Grassberger P. Estimating mutual information. Phys Rev E Stat Nonlin Soft Matter Phys. 2004;69: 066138. 15244698 10.1103/PhysRevE.69.066138

[149] Lange OF, Grubmuller H. Generalized correlation for biomolecular dynamics. Proteins. 2006;62: 1053–1061 16355416 10.1002/prot.20784

[150] Floyd RW. Algorithm 97: Shortest Path. Commun ACM. 1962;5: 345.

