# Supplementary figures and images for: Exploring Molecular Mechanisms of Paradoxical Activation in the BRAF Kinase Dimers: Atomistic Simulations of Conformational Dynamics and Modeling of Allosteric Communication Networks and Signaling Pathways
Source: PLoS One. 2016 Nov 18;11(11):e0166583. doi: 10.1371/journal.pone.0166583 (PMC5115767; doi:10.1371/journal.pone.0166583)

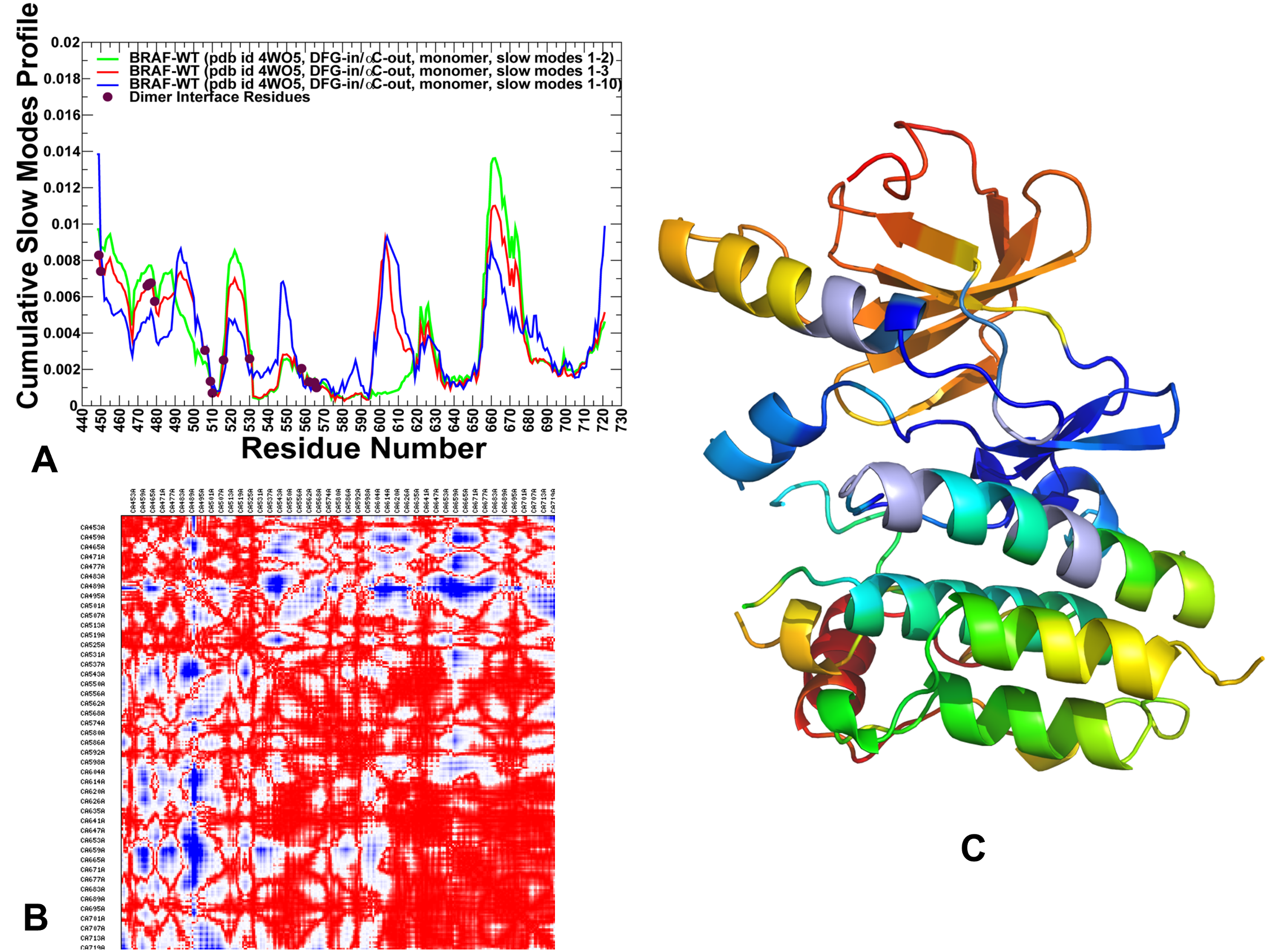

Supplement: S1 Fig — (A). The GMM-based conformational mobility profiles of the BRAF-WT monomer structure (pdb id 4WO5). The essential mobility of kinase residues is shown in the space of first two slowest modes (in green), the first three slowest modes (in red) and the first ten slowest modes (in blue). The position of the dimer interface residues in these profiles are highlighted in filled maroon-colored circles. (B) The inter-residue distance fluctuations map in the BRAF-WT is based on residue movements along the first three slow modes and derived using the ANM web server [105]. The large inter-residue fluctuations are shown in blue and small fluctuations are shown in red. (C) Structural maps of collective dynamics are based on fluctuations driven by the slowest three modes. The color gradient from red to blue indicates the increasing structural rigidity and refers to an average value over the backbone atoms in each residue. (TIF) [file pone.0166583.s001.tif]

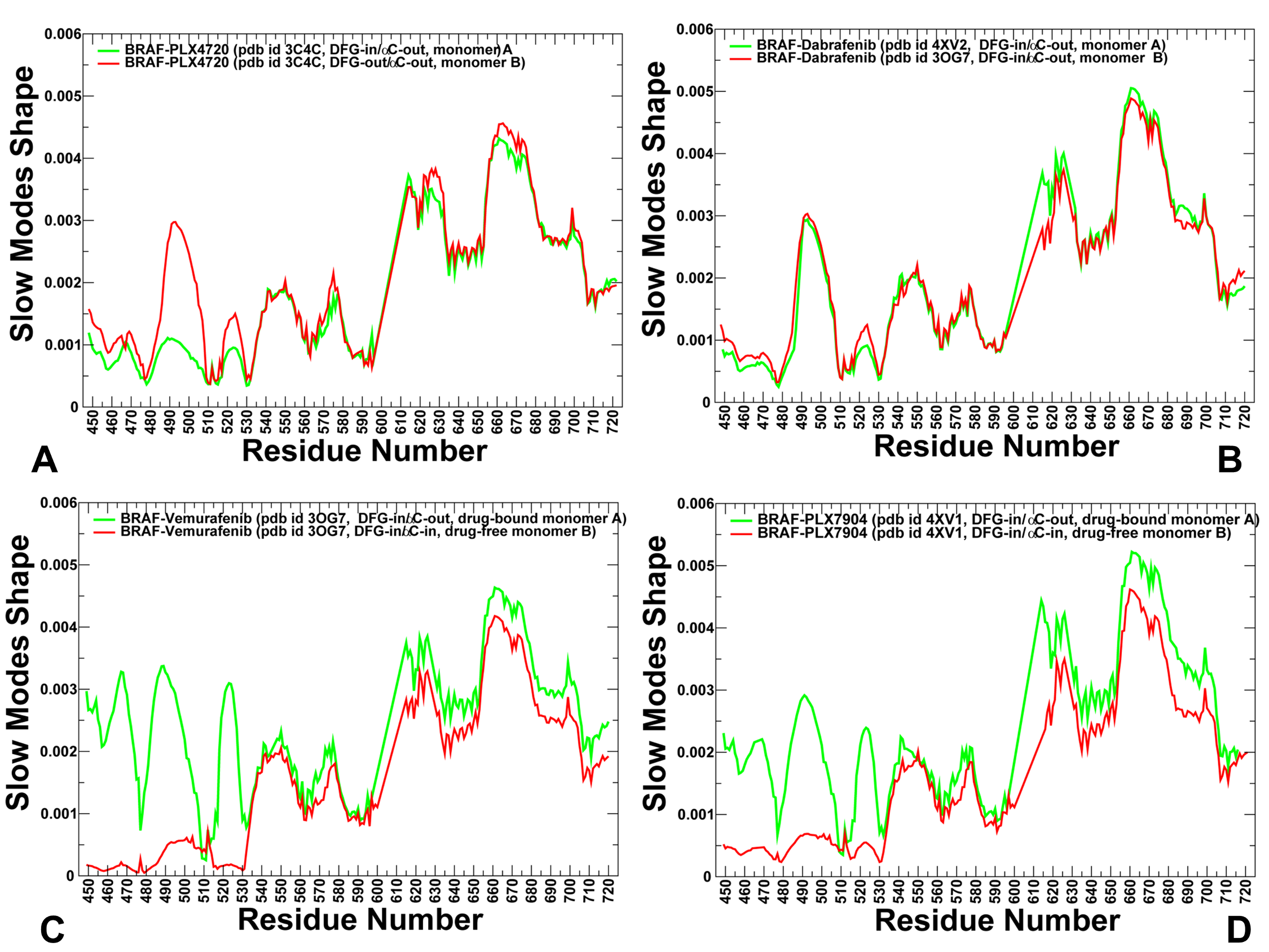

Supplement: S2 Fig — The GMM-based conformational mobility profiles along the three slowest modes are shown for the BRAF dimer complex with PLX4720 (pdb id 3C4C) (A), Dabrafenib (pdb id 4XV2) (B), Vemurafenib (pdb id 3OG7) (C), and PLX7904 (pdb id 4XV1) (D). The slow modes shapes are colored in green (first monomer) and red (second monomer). (TIF) [file pone.0166583.s002.tif]

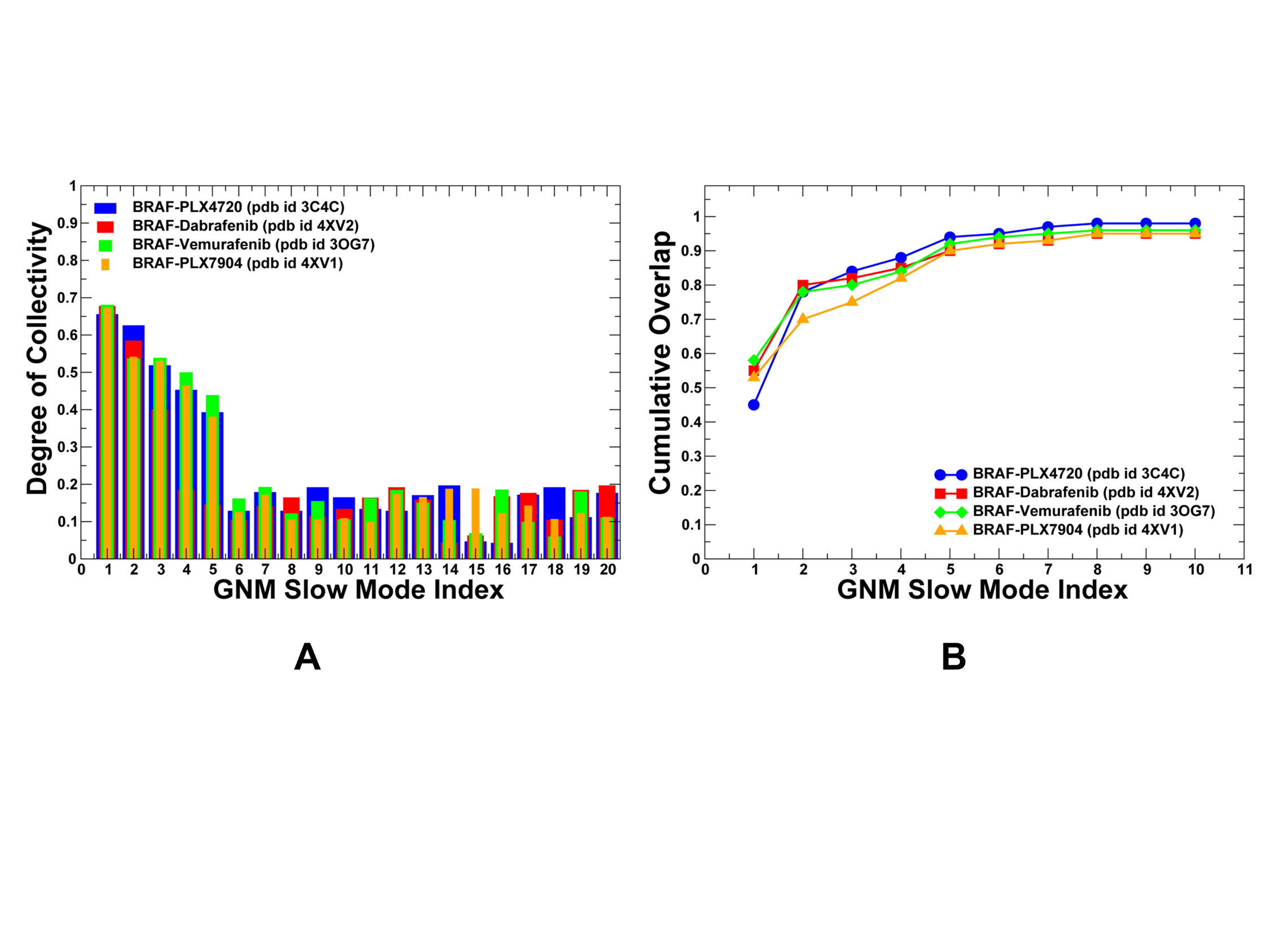

Supplement: S3 Fig — (A) The degree of collectivity of first 20 slow modes in the BRAF dimer structures is shown for the PLX4720-BRAF complex (in blue), Dabrafenib-BRAF complex (in red), Vemurafenib-BRAF complex (in green) and PLX7904-BRAF complex (in orange). High collectivity of the first five modes indicates that conformational movements in the BRAF structures can be described using a cumulative contribution of these slow modes. (B) Cumulative overlap of the GNM slow modes with the principal components describing conformational changes in the ensemble of BRAF crystal structures. This ensemble included all known BRAF dimer complexes with different classes of inhibitors as described in Fig 1. The softest five modes described ~90% of structural variations observed in the BRAF crystal structures. (TIF) [file pone.0166583.s003.tif]

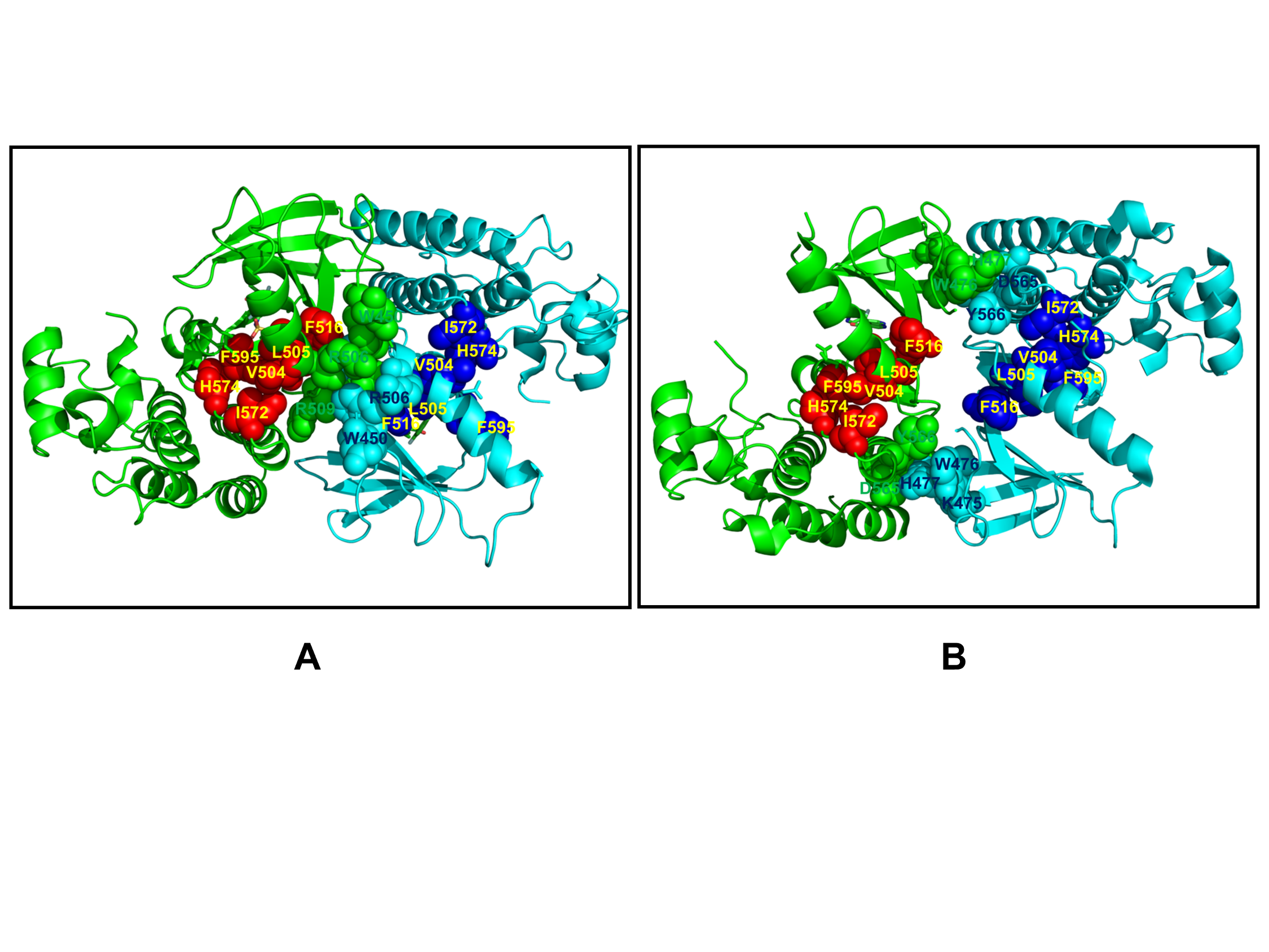

Supplement: S4 Fig — (A) Structural mapping of the central interface section. The first monomer is shown in green ribbons and the second monomer is shown in cyan ribbons. The residues from the central section of the dimer interface (R506, R509, and F516) are annotated and shown as green spheres (first monomer) and cyan spheres (second monomer). The R-spine residues (V504, L505, F516, I572, H574, and F595) are annotated and shown as red spheres (first monomer) and blue spheres (second monomer). (B) Structural mapping of the secondary interface regions. The interface residues in this region (K475, W476, H577, R558, R562, D565, and Y566) are annotated and shown as green spheres (first monomer) and cyan spheres (second monomer). (TIF) [file pone.0166583.s004.tif]
